# Enhanced Separation of Intact Proteins and Proteoforms by CZE‐MS Using Sulfobetaine‐Modified Poly(α‐L‐lysine)‐Based Multilayer Coatings for EOF Adjustment

**DOI:** 10.1002/pmic.70012

**Published:** 2025-07-25

**Authors:** Alisa Höchsmann, Henry Frick, Laura Dhellemmes, Laurent Leclercq, Philipp T. Kaulich, Andreas Tholey, Hervé Cottet, Norbert Schaschke, Christian Neusüß

**Affiliations:** ^1^ Faculty of Chemistry Aalen University Aalen Germany; ^2^ Faculty of Science University of Tübingen Tübingen Germany; ^3^ IBMM University of Montpellier CNRS ENSCM Montpellier France; ^4^ Systematic Proteome Research & Bioanalytics Institute for Experimental Medicine Christian‐Albrechts‐Universität zu Kiel Kiel Germany

**Keywords:** capillary coatings, capillary electrophoresis, CZE‐MS, polyelectrolyte multilayers, TD proteomics

## Abstract

Mass spectrometry–based top‐down protein analysis requires efficient separation. In the context of proteoform analysis, capillary zone electrophoresis (CZE) is very valuable. The resolution of two peaks in CZE can be increased when the absolute mobility of the counter‐directed electroosmotic flow (EOF) is close to the effective mobility of the analytes, resulting in a low apparent mobility of the analytes. The mobility of the EOF of highly efficient sulfobetaine‐modified poly(α‐L‐lysine) (α‐PLL) coatings changes depending on the number of modified side chains. Here, such coatings are used to selectively increase the peak resolution of proteoforms of model proteins and analytes in a complex protein sample (intact yeast protein extract). Whereas a high EOF system allows for the separation of proteins of a wide mobility range (complete proteome), lower EOF systems allow for a much better separation of proteins and proteoforms of low mobility, including those containing acidic post‐translation modifications (PTMs). This leads to the identification of 2.5 times more proteoforms by MS/MS experiments in the lower mobility range of the yeast proteome. The sulfobetaine‐modified α‐PLL coatings presented here exhibit a toolbox for highly resolved separation of proteins and proteoforms in targeted or untargeted top‐down protein analysis.

**Summary:**

- Sample complexity is one of the main challenges when analyzing a proteome on the proteoform level.
- In the course of this, capillary electrophoresis–mass spectrometry turned out to be an excellent tool because of its high‐performing separation, particularly for large molecules.
- Here, we present a method enabling the best possible separation due to efficient and EOF‐tunable coatings, allowing for flexible and dedicated selection of a range of proteins and proteoforms to be analyzed under ideal separation conditions.
- The high performance is demonstrated by the separation of proteoforms of common PTM‐rich model proteins as well as complex proteome samples.

**Abbreviations**
α‐PLL: poly(α‐L‐lysine)
AGP: α‐acid glycoprotein
BGE: background electrolyte
BSA: bovine serum albumin
FA: formic acid
HAc: acetic acid
IPA: isopropyl alcohol
LPA: linear polyacrylamide
PDADMAC: poly(diallyldimethylammonium chloride)
PSPSL*_x_*_%_
: PDADMAC‐PSS with last layer *X*%‐modified α‐PLL
PSPSP: five‐layered PDADMAC‐PSS coating
PSS: poly(sodium styrene sulfonate)
SMIL: successive multiple ionic polymer layers
TDP: top‐down proteomics
UPW: ultrapure water

## 1 Introduction

Mass spectrometry (MS) is an important tool for the characterization of proteins and their proteoforms [1]. In the context of the analysis of complex proteomes, efficient separation of proteoforms prior to MS is required [2, 3] in order to decrease co‐elution/co‐migration and, thus, reduce spectra complexity, resulting in an overall increased analysis depth. Here, capillary zone electrophoresis (CZE) is an excellent separation technique because of its ability to efficiently separate charge and size variants [4, 5, 6]. Furthermore, CZE provides high separation efficiency, flexible separation time, and requires low sample volumes [7, 8].

To minimize protein adsorption in CZE‐MS, capillaries are mostly coated with semi‐permanent or permanent capillary coatings [9]. Common coatings are either neutral, resulting in a suppressed electroosmotic flow (EOF), or cationic, where a reversed directed EOF is induced. For protein separation, one of the most commonly used neutral coatings is linear polyacrylamide (LPA) [10, 11, 12, 13, 14]. Separation with LPA coatings is characterized by high separation efficiencies and allows for sample preconcentrating of large sample volumes using dynamic pH junction [15].

In the case of cationic coatings, successive multiple ionic polymer layer (SMIL) coatings have proven to be advantageous because of their simple coating protocols, high migration time stability, and low protein adsorption [16, 17, 18, 19]. SMIL coatings are constructed by alternatingly flushing a polycation and polyanion through a fused silica capillary. Using cationic SMIL coatings, it is possible to reach almost one million plates per meter for the separation of proteins by CZE‐UV [20]. Another benefit of ionic coatings is, in general, the EOF strength that can be used as a tool to increase resolution when the effective mobility of the analytes is close to the reverse‐directed absolute mobility of the EOF. This has been demonstrated for various analytes such as peptides [21], proteins [22], and monoclonal antibodies [23]. Because the effective mobility of proteins can vary greatly, it is desired to have coatings varying in their EOF strength for different analytical challenges.

Ideally, instead of testing the EOF strength of various polycations, polymers are synthesized with a selectable EOF, a possibility that was accomplished in several studies. The polyanion poly(acrylamide‐co‐2‐acrylamido‐2‐methyl‐1‐propanesulfonate) (PAMAMPS) was used in a bilayer coating to analyze peptides by CZE‐UV with an anodic EOF [24]. Another coating is based on the cationic copolymer poly(acrylamide‐co‐(3‐acrylamidopropyl)trimethylammonium chloride) (PAMAPTAC), where the EOF strength changes depending on the monomer mixture. Initially, it was applied to the separation of hydrophobic drugs [25] and later applied to top‐down proteomics [26]. Another way to reduce the EOF of cationic coatings is the use of PEGylation to modify the polycation poly(allylamine hydrochloride) [27]. Similarly, the net charge of poly(α‐L‐lysine) (α‐PLL) can be reduced by partially modifying the positively charged side chains with a zwitterionic sulfobetaine functionality [28]. This results in reduced absolute EOF mobility, while the separation efficiency of these novel coatings is as high as other SMIL coatings [28].

In this study, such cationic SMIL coatings are for the first time used for the highly efficient separation of proteins and proteoforms by CZE‐MS. α‐PLL‐based coatings partially modified by a zwitterionic sulfobetaine functionality assure high peak resolution because of the high separation efficiency and adjustable mobility of the EOF depending on the degree of zwitterionic modification of the polylysine coatings. This increase in resolution in defined mobility frames is demonstrated for best separation of proteoforms of several proteins, including α‐1‐acid glycoprotein (AGP), fetuin, bovine serum albumin (BSA), and casein. Moreover, the generalized increase of protein resolution in certain mobility ranges is demonstrated by top‐down CZE‐MS/MS experiments of an intact yeast protein extract.

## 2 Materials and Methods

### 2.1 Materials

Poly(diallyldimethylammonium chloride) (PDADMAC) (Mw 400,000–500,000 g/mol), poly(sodium styrene sulfonate) (PSS) (Mw 70,000 g/mol), hydrofluoric acid (40% (v/v)), ammonium acetate, and sodium hydroxide were acquired from Merck KGaA (Darmstadt, Germany). Poly(α‐L‐lysine) hydrochloride (α‐PLL; average Mw 66 × 103 g/mol) with a polydispersity index between 1.0 and 1.2 was purchased from Alamanda Polymers (Huntsville, USA). Isopropyl alcohol (IPA), formic acid (≥98%, FA), ammonia solution (25%), and acetic acid (HAc) were obtained from Carl Roth GmbH & Co. KG (Karlsruhe, Germany). All solutions were prepared with ultrapure water (UPW, 18 MΩ*cm at 25°C, SG Ultra Clear UV from Siemens Water Technologies, USA).

Carbonic anhydrase from bovine erythrocytes, myoglobin from equine skeletal muscle (purity ≥ 95%), ribonuclease A from bovine pancreas (purity ≥ 60%), β‐lactoglobulin A from bovine milk (purity ≥ 90%), lysozyme from chicken egg white (purity ≥ 90%), fetuin from fetal bovine serum, casein from bovine milk, AGP from human plasma, BSA, USP mAb003 (lot: F12980), and 4‐(2‐hydroxyethyl)‐1‐piperazineethanesulfonic acid (HEPES) were purchased from Sigma‐Aldrich (Steinheim, Germany). All proteins were diluted in water to a concentration of 4 mg/mL that was stored in the freezer at −20°C. Before measurements, proteins were mixed and diluted to a final concentration of 200 µg/mL (1 mg/mL in the case of fetuin, BSA, and AGP) in 50 mM ammonium acetate adjusted first to pH 8 with ammonia. Subsequently, 100 mM HAc was added to the sample before the sample was heat treated at 37°C using a Thermomixer (Eppendorf, Wesseling‐Berzdorf, Germany). To prevent sample precipitation, casein was solved and diluted in 1 M NH_3_.

Whole‐cell protein extract (intact) from *Saccharomyces cerevisiae* cells (Promega, Walldorf, Germany) was diluted to a final protein concentration of 1 mg/mL in 50 mM ammonium acetate (pH 8); the solution was centrifuged at 10,000 rpm for 90 s and then split into aliquots of 25 µL for storage. Until use, it was stored at −80°C. Before measurement, HAc was added to a concentration of 100 mM to precipitate analytes that were not solvable under acidic background electrolyte (BGE) compositions. The sample was vortexed and then centrifuged at 14,500 rpm for 5 min. Ten microliters of the supernatant was transferred to a fresh insert and centrifuged again at 14,500 rpm for 5 min prior to measurement. During measurement, the CE sample tray was cooled to 4°C using a Lauda ecoline external cooling system.

### 2.2 Modified Poly(α‐L‐lysine) Synthesis

Synthesis of the modified α‐PLL has been described in more detail previously [28, 29]. Briefly, 3‐((Carboxymethyl)dimethylammonio)propane‐1‐sulfonate was dissolved in H_2_O/MeOH (1:1, v:v) and deprotonated with *N*‐methylmorpholine (2 equiv.). The solution of the deprotonated zwitterion and the coupling reagent 4‐(4,6‐dimethoxy‐1,3,5‐triazin‐2‐yl)‐4‐methylmorpho‐linium chloride (1:1 molar ratio) were added to an α‐PLL solution (dissolved in H_2_O:MeOH (1:1,v:v), concentration 10 mg/mL) in the desired stoichiometric ratio under vigorous stirring. The reaction proceeded overnight at room temperature, followed by dialysis against 0.5 M sodium perchlorate for 48 h. After lyophilization for 48 h, the obtained polymer was a colorless solid. The degree of functionalization was determined by ^1^H NMR from the ratio of the integrals of the signals of the α‐CH protons of the Lys residues of α‐PLL and the signals of the zwitterionic moieties [28]. The modified α‐PLL is shown in Figure S1.

### 2.3 Capillary Electrophoresis

CZE‐UV was performed with a CE 7100A system from Agilent Technologies (Waldbronn, Germany). Bare fused silica capillaries used as a separation capillary/sheath liquid capillary with 50/100 µm inner diameter and 360/240 µm outer diameter were obtained from Polymicro Technologies (Phoenix, USA).

SMIL capillaries were coated according to the protocol of Dhellemmes et al. [18]. Briefly, capillaries were flushed with 1 M NaOH for 10 min, UPW for 5 min, and 20 mM aqueous HEPES solution (pH 7.4) for 10 min. To create SMIL layers, the capillary was alternately flushed with polycation and polyanion solution (3 g/L in 20 mM HEPES solution at pH 7.4) for 7 min with intermediate flushing with HEPES solution for 3 min until a total of 5 layers was reached. After a waiting step of 5 min, the capillary was flushed with UPW for 3 min and background electrolyte (BGE) for 10 min. Before each measurement, the capillary was flushed for 3 min with BGE. All coating steps and flushing steps were performed at 2 bar when using 60 cm capillaries and 6 bar when using 180 cm capillaries. If not otherwise stated, the injection was 70 s, 33 mbar for the 60 cm capillary (5% capillary volume) and 210 s, 99 mbar for the 180 cm capillary (5% capillary volume). For all separations, 2 M HAc was used as the BGE, apart from the mAb where 4 M HAc was used.

When recoating was performed, the following protocol was utilized: capillary flushing for 5 min with UPW, 3 min with HEPES solution, 7 min with polycation, 3 min with HEPES solution, 3 min with UPW, and 10 min with BGE. In the case of all coatings, lower layers (1–4) were PDADMAC (P) and PSS (S). When the last layer was PDADMAC, the coating is referred to as PSPSP. When the novel zwitterionic modified α‐PLL (L*_X_*_%_) is used, the coating is termed PSPSL*_X_*_%_ with *X* referring to the percentage of modified α‐PLL side chains. In between measuring days, the SMIL capillaries were stored in BGE. Data processing for CZE‐UV data and calculation of the full‐width half maximum (FWHM) of peaks during MS measurements were performed using CEval 0.6h9 [30] (available at: https://echmet.natur.cuni.cz/).

### 2.4 Mass Spectrometry

For CZE‐MS coupling, a CE 7100A system was coupled to an Orbitrap Fusion Lumos mass spectrometer (Thermo Fisher Scientific, San Jose, USA) using the *nanoCEasy* interface [31]. For MS detection, the tip of the CZE capillary was etched with hydrofluoric acid to an outer diameter of roughly 80–100 µm. Emitters with a 30‐µm tip were obtained from BioMedical Instruments (Zöllnitz, Germany). IPA:UPW (50:50) + 0.5% FA was used as the sheath liquid. The spray voltage for the nanoCEasy interface was set to 2000 V. All yeast sample data and casein data were acquired in positive mode using a mass range of 400–2000 *m/z* and a resolution of 120,000. Fragmentation experiments were performed using collision‐induced dissociation (CID) with a normalized collision energy of 30%. Further MS parameters can be found in Table S1.

All raw data and database search results have been deposited to the ProteomeXchange Consortium [32] via the PRIDE partner repository with the dataset identifier PXD061793.

### 2.5 Data Evaluation

In order to determine the intact proteoform masses, high‐resolution (i.e., isotopically resolved) MS1 data were deconvolved using Freestyle 1.8 (Thermo Fisher Scientific, default settings, minimum detected charge 2, charge range 2–50). Not isotopically resolved mass spectra were analyzed using Intact Mass 3.4 (Protein Metrics). Data visualization was done using JMP Pro 16.1.0.

For proteoform identification, ProSightPD (v4.2) within Proteome Discoverer (v3.0) was utilized. The raw fragmentation data were deconvolved using the High/High cRAWler (standard grouping with maximal retention time difference of 0.5 min and precursor tolerance of 0.2 *m/z*, Xtract deconvolution) and searched against a protein database containing yeast proteins (only reviewed proteins downloaded from UniProt as an XML file [including all known modifications], taxon‐id 559292, release 2023_01). Proteoform identification was performed using the Annotated Proteoform Search and Subsequence Search nodes (precursor and fragment mass tolerances: 10 ppm) with default settings and the identifications were filtered using a context‐dependent 1% false‐discovery rate [33].

## 3 Results and Discussion

### 3.1 Resolution and EOF

Increasing the resolution between two peaks has many advantages. For the detection of some analytes, it is even required, like when measuring proteoforms with similar masses that cannot be resolved by MS (e.g., deamidated proteoforms), isomers, or for better quantitation, especially in the context of low‐abundance proteoforms that would be suppressed during the electrospray ionization process by higher abundant proteoforms. The resolution in CZE can be described by Equation (1):

(1) $$R=0.25\frac{\Delta \mu_{e}}{\left|\mu_{\mathrm{EOF}}+{\bar{\mu }}_{e}\right|}\sqrt{N}$$

and is therefore depending on the effective mobility difference between two analytes ($\Delta \mu_{e}$), the mobility of the EOF ($\mu_{\mathrm{EOF}}$), the mean effective mobility of two analytes (${\bar{\mu }}_{e}$), and the number of theoretical plates (*N*). When the same BGE is used, $\Delta \mu_{e}$ and ${\bar{\mu }}_{e}$ are constant, whereas *N* and $\mu_{\mathrm{EOF}}$ can be influenced by the capillary coating.

One way to gain high resolution is using capillary coatings in which the separated analytes have a high separation efficiency and low protein adsorption. In a previous publication, Dhellemmes et al. [28] showed that excellent plate heights (i.e., a high separation efficiency) can be obtained for the zwitterionic modified α‐PLL coatings (compare zwitterion 1), when used as the last layer on cationic PDADMAC and anionic PSS‐SMIL coatings. The plate heights were in a range similar to the five‐layer PDADMAC‐PSS (PSPSP) coating, one of the most efficient cationic SMIL coatings [18, 34].

**FIGURE 1:**
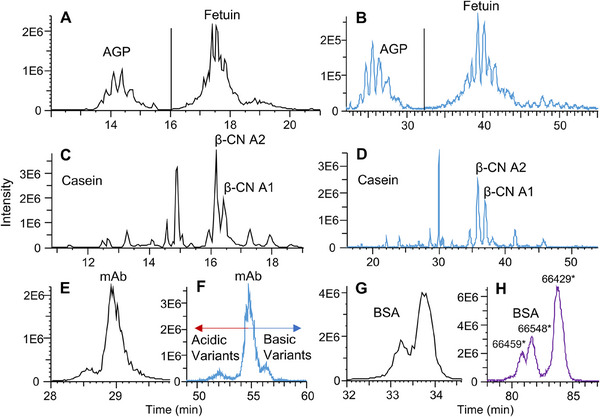
Electropherogram of selected biomolecules A and B) AGP and fetuin, C and D) casein, E) and F) USP mAb003, and G and H) BSA (*mass obtained by Protein Metric), separated on different coatings PSPSP (black, A, C, E, G), PSPSL_51%_ (purple, H), PSPSL_71%_ (blue, B, D, F). More detailed information on the separation conditions can be found in Table S2 and the Materials and Methods section.

Another way to increase the resolution is to decrease the sum of the effective mobility of the analyzed cations and the counter‐directed absolute mobility of the EOF. In the case of the zwitterionic modified α‐PLL coating, the absolute EOF mobility can be adjusted depending on the amount of lysine side chains that are modified with the zwitterionic sulfobetaine functionality. In general, the more lysine side chains are modified, the lower the net charge of the modified α‐PLL and the lower the absolute EOF mobility. In a previous publication [28], we determined for a 2 M HAc BGE, an EOF in the range of 25–50 TU (TU = Tiselius unit, 1 TU = 10^−9^ $\frac{m^{2}}{V.\;s}$) depending on the degree of modification. On the basis of this previous work, we selected the following coatings: PSPSP, PSPSL_51%_, and PSPSL_71%,_ with an absolute EOF mobility of 45.2, 36.9, and 28.8 TU, respectively, to achieve the best separation performance for the proteoforms of various model proteins as summarized in Figure 1. All effective mobilities in the following paragraph were calculated based on the method described in the Supporting Information.

AGP is a glycoprotein with a large number of different glycoforms, including sialic acids. It has a low isoelectric point (pI 2.7) [35] and a low effective mobility (7.9–12.8 TU) in 2 M HAc. The separation using the PSPSL_71%_ coating (Figure 1B) is improved compared to the PSPSP coating (Figure 1A). Due to the low effective mobility of AGP, an even better resolution is expected for a cationic coating with an even lower EOF (higher degree of zwitterionic modification) or a neutral coating with a similar separation efficiency. For fetuin (effective mobility 14.6–20.0 TU), the PSPSL_71%_ coating (Figure 1B) also achieves higher resolved peaks of the glycoforms compared to the PSPSP coating (Figure 1A). Dozens of proteoforms can be detected (Figure S2) based on baseline separation of proteoforms differing in one phosphate and sialic acid, respectively, as well as clear separation of proteoforms differing just in one antenna (HexHexNAc), as shown in Figure S3. For AGP an increase of 1.7 in resolution was achieved, whereas for fetuin an increase of 2.2 in resolution was achieved when comparing the PSPSL_71%_ coating to the PSPSP coating (Table S3). Compared to the separation found in the literature on a neutral coating (UltraTol Dynamic Precoat LN, BGE: 2 M HAc), the separation of fetuin has clearly improved [36]. Similarly, the best separation of proteoforms of β‐casein (*µ*_e_ = 14.3–16.2 TU) (Figure 1D) is achieved using the PSPSL_71%_ coating compared to the PSPSP coating (Figure 1C). Again, baseline separation of proteoforms differing in one phosphorylation is achieved (Figures S4 and S5). Additional information on α‐casein (µ_e_ = 20.2–21.9 TU) can be found in the Supporting Information (Figure S4, Table S4). Compared to the CZE‐MS separation found in the literature (hydroxypropyl cellulose coating, BGE: 2 M HAc) [37], the separation has improved. In the case of the USPmAb003, the advantage of medium EOF coatings was already shown previously [23] for a diethylaminoethyl‐dextran‐PSS coating. A similar resolution is obtained here for the PSPSL_71%_ coating (Figure 1F). Compared to a neutral coating [23] or the PSPSP coating (Figure 1E), the separation has strongly improved. The effective mobility of the three BSA variants is about 30 TU and cannot be separated by the PSPSL_71%_ coating. Instead, the separation by the PSPSL_51%_ (Figure 1H) coating improves separation, that is, separates an additional peak compared to the PSPSP coating (Figure 1G).

In conclusion, it is shown how the separation of proteoforms for a range of complex model proteins can be improved by selecting the appropriate coating with a suitable EOF. The mobility of the EOF is adjustable by the degree of zwitterionic modification of α‐PLL as the last layer in a five‐layer PSPSL*_X_*_%_ approach. Depending on the desired mobility of the EOF, additional coatings with different degrees of modification could be synthesized. While the resolution could be further improved by using coatings with an even lower EOF, this results in an increase in migration time. In general, when decreasing the EOF strength as demonstrated here, the increased peak resolution always comes at the downside of an increased migration time. This downside can be partially overcome by increasing the separation voltage as will be discussed more in detail later.

### 3.2 Preconcentration and Repeatability

Injecting large sample volumes can help to increase the typically low concentration sensitivity of CZE when analyzing proteins and proteoforms in biological samples. Here, a sample buffer of 50 mM ammonium acetate with 100 mM acetic acid was used (pH 4.5). To show the functionality of sample preconcentration on cationic capillary coatings like SMIL coatings, the PSPSP capillary was filled with different sample volumes ranging from 0.5% to 20% of the total capillary volume, and the separation efficiency for the standard mixture of five proteins was compared (Figure S6). Hereby, the separation efficiency decreases by a factor of 1.0–1.6 for carbonic anhydrase, ribonuclease A, β‐lactoglobulin A, and lysozyme and 2.5 for myoglobin when comparing an injection volume of 0.5%–5% of the total capillary volume. A five‐fold increase in peak intensity was obtained (10‐time diluted sample results in a two‐times lower intensity). Thus, an injection volume of 5% was used for the following experiments of analyzing a yeast protein extract as a good compromise between increased signal intensity and decreased separation efficiency.

**FIGURE 2:**
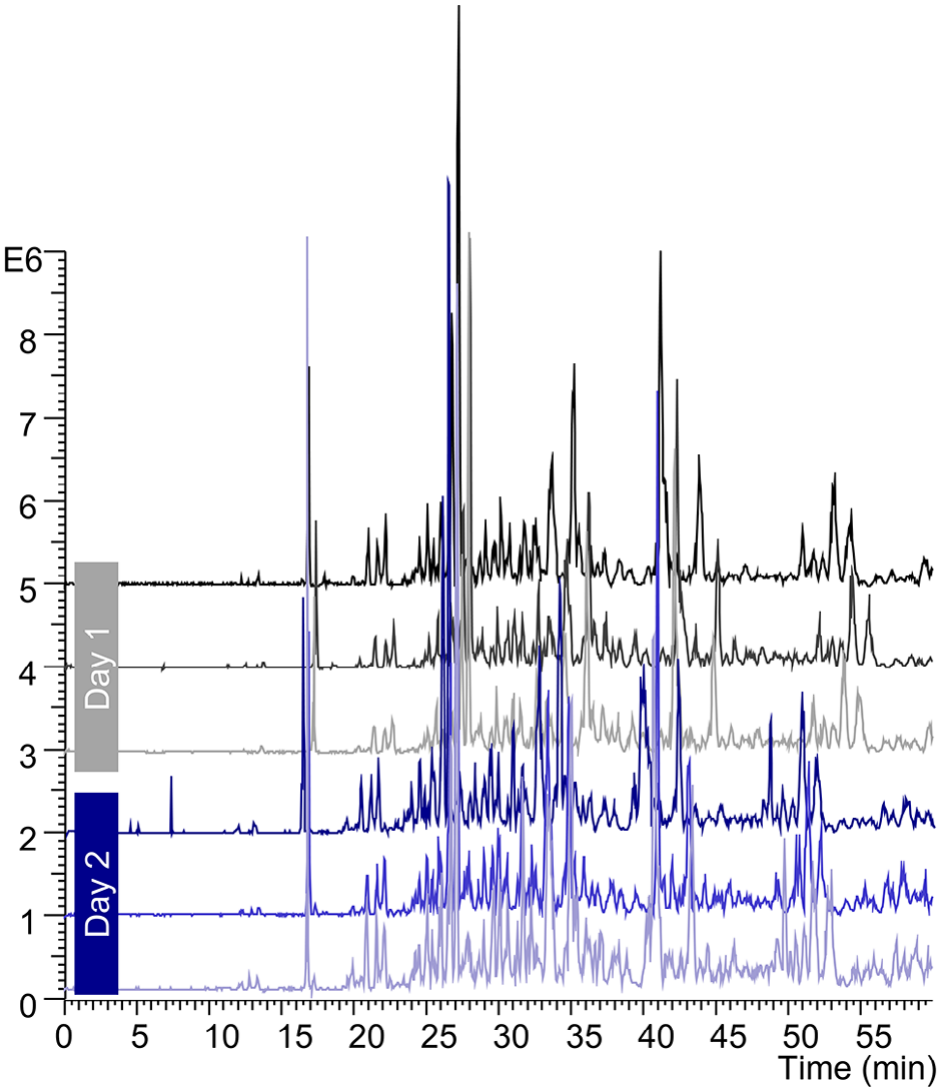
Repeatability of yeast protein extract measurements using a PSPSL_71%_ capillary. Top: first measurement, bottom last measurement. Measurements over 2 days (*n* = 2×3) with recoating in between each measurement. 60 cm capillary, −10 kV separation voltage, injection volume: 5% of the total capillary volume. Other parameters can be found in the Materials and Methods section.

To show the repeatability and long‐term stability of the PSPSP‐SMIL capillaries for the analysis of complex protein samples, a yeast protein extract and the standard protein mixture were measured alternately by CZE‐UV. In these initial experiments, the protein peaks of the standard protein mixture became broader over time, and migration times increased (Figure S7). Although the impact is less noticeable for the first several measurements, the effect continues to increase over time. Ribonuclease A is particularly strongly impacted, with the peak intensity already decreasing for the second measurement and the peak completely disappearing after the fourth measurement. When analyzing only standard proteins, the separation performance is stable as described previously [18]. The gradual decrease of EOF could be induced by either the last layer of PDADMAC being unstable and being gradually removed or, more likely, by negatively charged matrix molecules from the yeast protein extract, irreversibly binding to the positively charged surface and thereby altering the coating properties. A way to prevent the deterioration of the coating is to recoat the capillary. The recoating protocol takes 30 min and can be performed without disassembling the CZE‐MS setup due to the valving functionality of the nanoCEasy interface [38]. A recoating after every measurement results in an overall good migration time repeatability for the CZE‐UV experiments (relative standard deviation [RSD] 0.03% over five proteins, *n* = 7) and stable performance with minimal differences in FWHM (RSD 2.4% over five proteins, *n* = 7) with the yeast protein extract measured in‐between (Figure S8). At the same time, the previously low abundant RNase A peak is retained at its initial abundance and shape. For CZE‐MS, the repeatability of the migration time for the standard protein mixture is 0.8%–1.3% (RSD, *n* = 4). In Figure 2, the intraday and interday repeatability of measuring the yeast protein extract on a PSPSL_71%_ capillary is shown, where the capillary is recoated in between every measurement, and the capillary is filled to 5% with sample. For the PSPSP coating, the intraday migration time repeatability ranges between 1.1% and 2.6% (RSD, *n* = 5, 9 low mobility analytes + 8 high mobility analytes), whereas for the PSPSL_71%_ coating, the intraday migration time repeatability was 1.3%–1.7% (*n* = 3, 9 low mobility analytes) and the interday migration time repeatability was 1.6%–3.1% (*n* = 3 repeats x 2 days, 9 low mobility analytes). The repeatability of the absolute mobility of the EOF was 1.0%, 1.1% and 1.2%, respectively. Because the deviations in the migration times are mainly caused by deviations in the EOF, a larger deviation for later migrating analytes is observed.

### 3.3 Intact Yeast Proteome Sample

**FIGURE 3:**
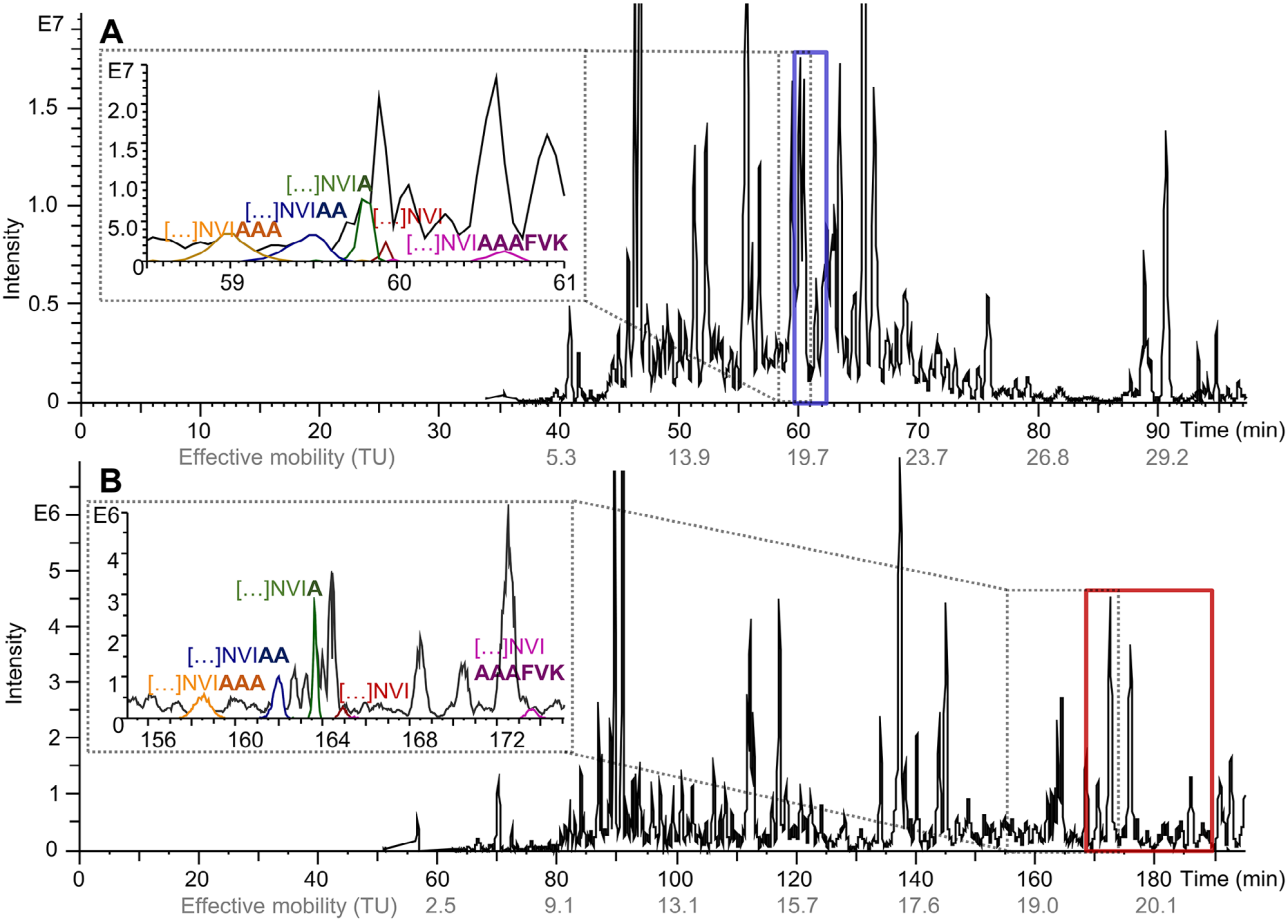
Comparison of the separation of the yeast protein extract for the high (A, PSPSP) and medium EOF (B, PSPSL_71%_) coating. Deconvoluted Base Peak Electropherogram in black. dashed insert: Separation of selected truncated forms (different colors, Table S9) of Enolase 2. Solid insert: Analytes with mobilities between 19.5 and 20.5 TU, which are used later for Figure 5B. 180 cm capillary length, −30 kV separation voltage. Other parameters can be found in the Materials and Methods section.

In order to investigate the performance of the presented separation system for a complex protein sample, the intact yeast protein extract was measured on a PSPSL_71%_ in comparison to the separation on a PSPSP capillary. The effective mobilities and absolute mobility of the EOF were calculated as described in the Supporting Information (Section 5). The PSPSP coating (*µ*_EOF_ = 48.0 TU) allows for the separation of most analytes (*µ*_e_ < 31 TU) in a time frame of 100 min when using a 180 cm capillary, a 2 M HAc BGE, and −30 kV separation voltage (Figure 3A). In comparison, when separating analytes using the PSPSL_71%_ coating (Figure 3B), (*µ*_EOF_ = 28.7 TU), only analytes with an *µ*_e_ < 21 TU were separated within 200 min.

An increase in resolution for the detected low‐mobility proteins is observed (Figure 3) for the PSPSL_71%_ coating compared to the PSPSP coating as expected based on Equation (1). This is exemplified in the inserts of Figure 3 for several forms of truncated enolase 2 (identified via MS/MS spectra). Compared to the separation on the PSPSP capillary (dashed insert, Figure 3A), the peak resolution on the PSPSL_71%_ coating (dashed insert, Figure 3B) increases, allowing for additional baseline separated peaks in between the enolase 2 forms. The separation of truncated enolase 2 fragments shows the separation power of CZE, with forms differing by one uncharged amino acid (alanine) being baseline separated from each other due to the increase in migration time for the smaller proteoforms. Similarly, the higher charged, although larger proteoform, migrates significantly later. When comparing the same separations on a 60 cm capillary and a 180 cm capillary, the resolution of the peaks decreases for both types of coating (Figure S11) for the shorter capillary, which could be influenced by the preconcentration method and the shortened separation pathway. Additionally, a decrease in signal intensity can be observed for the PSPSL_71%_ capillary compared to the PSPSP coating; this should be expected as faster migrating analytes typically result in narrower peaks while maintaining similar peak areas.

**FIGURE 4:**
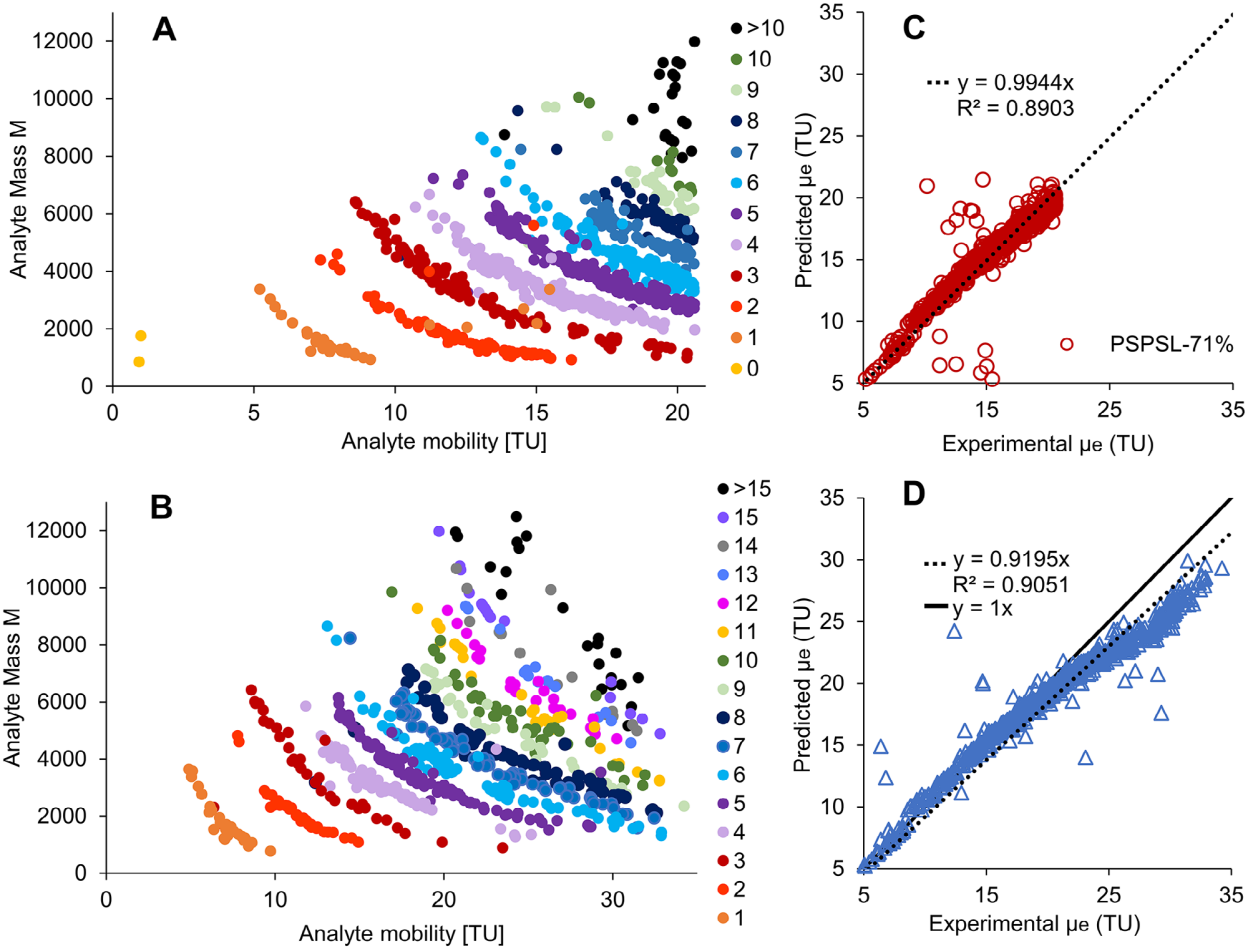
Correlations between (A, B) proteoforms’ experimental effective mobilities and their mass (net charge of the analytes visualized in different colors) and (C, D) the predicted and experimental effective mobilities of the proteoforms. Analytes were identified using ProSightPD, and net charge is obtained by counting basic amino acids and the N‐terminus as +1 (when not modified, e.g., acetylation) and modifications like phosphorylation as −1. Separation using 180 cm capillaries and −30 kV with (A, C) PSPSL_71%_ coating and (B, D) PSPSP coating. Other parameters are found in Materials and Methods. Experimental analyte mobility is obtained by the method described in the Supporting Information, whereas predicted analyte mobility is obtained by Equation (2).

In order to evaluate the separation and the MS/MS identification, the effective mobility of all identified analytes is plotted against their deconvoluted mass, annotating the net charge (*Q* = number of basic amino acids + 1) in different colors for the PSPSL_71%_ coating in Figure 4A and for the PSPSP coating in Figure 4B.

In Figure 4A,B, proteins with the same net charge are grouped along lines as previously shown for peptides [39]. The effective mobility increases for proteoforms with a lower mass or higher positive charge. Based on Figure 4A, it seems that the PSPSL_71%_ coating, which allows the analysis of proteoforms with effective mobilities up to 21 TU, is more suited for smaller proteoforms. However, as shown for fetuin, casein, and AGP (Figure 1), it is also suited for these larger proteins bearing PTMs, significantly reducing charge and thus mobility of the protein. This makes PSPSL_71%_ attractive for the separation of proteins with acidic PTMs such as phosphorylation, glycosylation, and acetylation (reduction of basic functionality).

The relationship between the net charge, the mass, and the mobility allows the prediction of the effective mobility of peptides and proteins in open‐tubular CZE using Equation (2) [40, 41]:

(2) $$\mathrm{Predicted}\;\mu_{e}=5\times 100\times \frac{\ln(1+0.35Q)}{M^{0.411}}$$

with *M* being the mass of the proteoform and *Q* being the net charge of the proteoform (counting basic amino acids and the N‐terminus as +1 [when not modified, e.g., acetylation] and modifications like phosphorylation as −1) and 5 being the prefactor for 5% HAc [41], albeit here 2 M HAc (11.4%) is used as the BGE.

In Figure 4C,D, the experimental *µ*_e_ of the sample (Supporting Information: Section 5) is plotted against the predicted effective mobility value from Equation (2). There is a linear correlation between both effective mobilities for most analytes. Analytes with higher mobility values have higher mobilities than predicted and no longer follow the linear slope perfectly. Still, with Equation (2) being able to predict the effective mobility of most analytes correctly, a high success in correct identification is likely. For the range between 5 and 20 TU, 94.4%/96.1% (PSPSP/ PSPSL_71%_) of the values fit the slope *y* = 1*x* (± 10%).

For the 180 cm capillary with PSPSP and PSPSL_71%_ coating, a total average number (*n* = 3) of 683 and 978 proteoforms from 178 and 214 proteins were identified, respectively. In agreement with other TDP studies, the vast majority of the identified proteoforms are smaller than 20 kDa [42]. Moreover, similar to the findings of Zhao et al. [12], a high percentage of truncated yeast proteoforms were observed. The bias toward the identification of small proteoforms can be explained by the decreased sensitivity of large proteoforms due to the isotopic‐ and charge‐dilution effect (i.e., the splitting of the signal across multiple charge states and isotopic peaks) [43].

**FIGURE 5:**
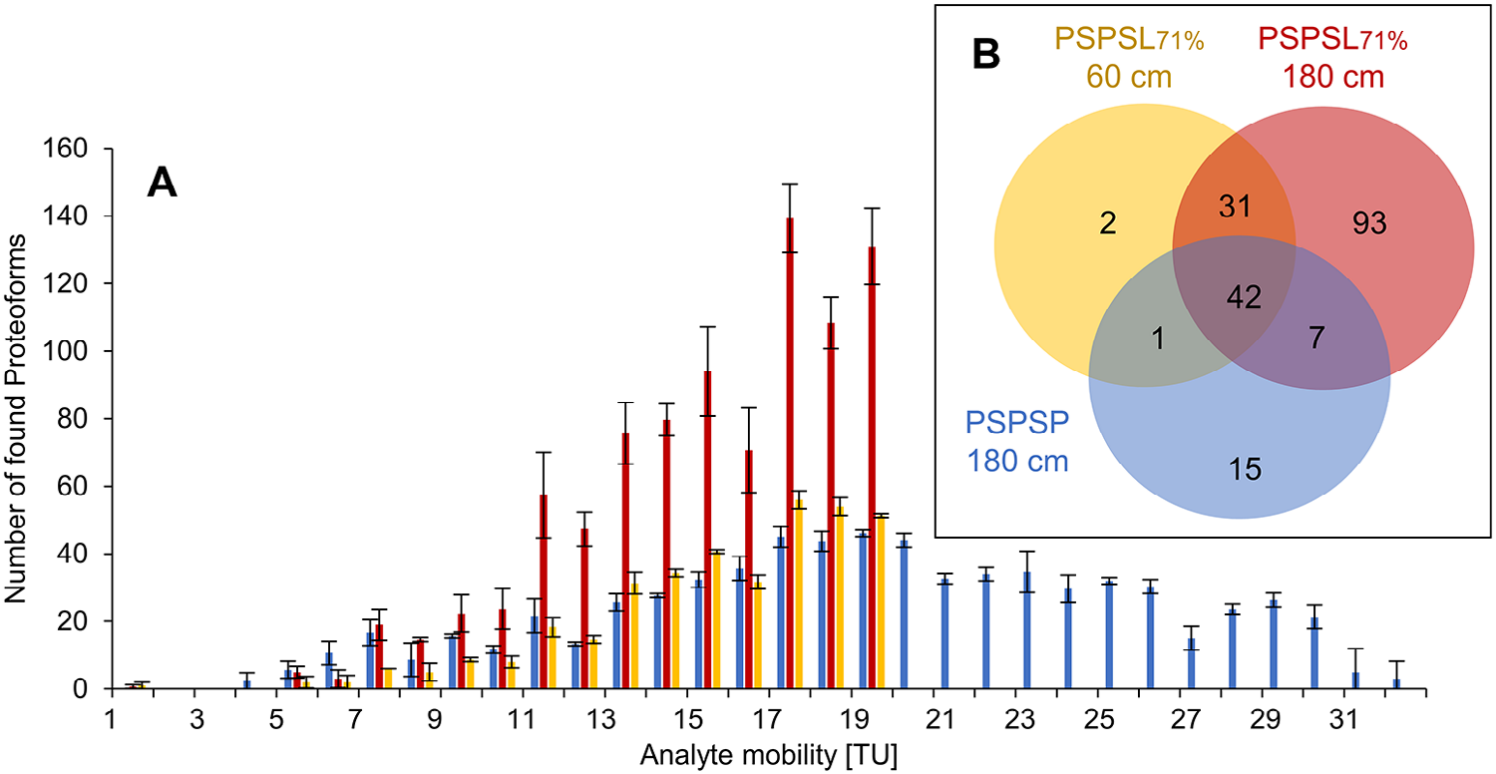
Number of proteoforms found for different effective analyte mobilities. Comparison of two PSPSL_71%_ capillaries with a length of 60 cm (yellow) and 180 cm (red) and a PSPSP capillary with a length of 180 cm (blue). A) The number of proteoforms identified over the whole effective mobility range (*n* = 3, SD for each bin, with each bin being a mobility range of 1 TU) and B) the number of unique and overlapping proteoforms with effective mobility between 19.5 and 20.5 TU found in at least one of three repeat measurements.

When comparing proteoforms within the same mobility frame (i.e., <21 TU) on both 180 cm capillaries, for the PSPSL_71%_ capillary, an average of 978 proteoforms were identified, whereas for the PSPSP capillary, only 387 proteoforms could be found. If analytes with higher mobilities (>21 TU) are considered, an additional 296 analytes were identified using the PSPSP coating that could not be identified with the PSPSL_71%_ coating due to their high analyte mobility. This can be seen in Figure 5A as well, where the total amount of proteoforms found per mobility frame is visualized.

Especially for the 180 cm PSPSL_71%_ coating for the mobilities that can be separated, the number of identified proteoforms is a lot higher compared to the PSPSP coating or the shorter capillary. In the case of the 60 cm PSPSL_71%_ coating, the number of proteoforms is much lower compared to the 180 cm PSPSL_71%_ coating and only slightly higher than for the PSPSP coating. This was expected based on the lower resolution for shorter capillaries and shows that increasing the capillary length is another important tool to increase the number of proteoforms. Thus, the increase in time is the key to increasing the number of detected proteoforms in top‐down experiments, which can be performed by both using longer capillaries and by reducing the cationic EOF here (PSPSL_71%_ instead of PSPSP coating).

In order to demonstrate the increased number of analytes to be separated, the peak capacity was calculated (*k* = *t*/4*σ*), with 4*σ* based on the mean FWHM from 10 peaks later used for resolution calculation (Section 3.4), and *t* is the time range between the same first (*M* = 3374 Da) and last analyte (*M* = 2714 Da) found in all three repeats for all three capillaries. The 180 cm PSPSL_71%_ capillary has a peak capacity of 149 ± 4 (SD, *n* = 3), whereas the 60 cm PSPSL_71%_ capillary has a peak capacity of 88 ± 5 (SD, *n* = 3). In comparison, the 180 cm PSPSP capillary has a peak capacity of 61 ± 5 (SD, *n* = 3) for this mobility range and a peak capacity of 157 ± 12 (SD, *n* = 3) when the whole mobility range is considered.

As previously described, cationic SMIL coatings provide the best separation for a mobility range close to their absolute EOF mobility, that is, the last migrating analytes. Therefore, the number of analytes with effective mobility between 19.5 and 20.5 TU were compared for the PSPSL_71%_ coatings (60 and 180 cm) and the PSPSP coating (180 cm). Figure 5B shows the number of proteoforms identified based on the MS/MS. For the described mobility frame (Δ*µ* = 1 TU) and the long capillary (180 cm), the PSPSL_71%_ coating allows for the detection of 173 proteoforms, with the majority being unique, whereas the PSPSP coating enabled the detection of 65 proteoforms. Also, the 60 cm PSPSL_71%_ capillary allows for a smaller number of proteoforms (76). This again highlights the potential of lower EOF coatings to selectively increase the separation in certain mobility frames, which allows for increased separation of analytes from another while more MS/MS spectra can be obtained over the mobility frame due to the increased time and better peak resolution.

### 3.4 Calculation of Optimized Resolution

As of now, only two coatings were the main focus of this study (PSPSP with a high EOF and PSPSL_71%_ with a medium EOF), but a major advantage of the zwitterionic modified α‐PLL coatings is the possibility to synthesize for any kind of low EOF mobility [28].

**FIGURE 6:**
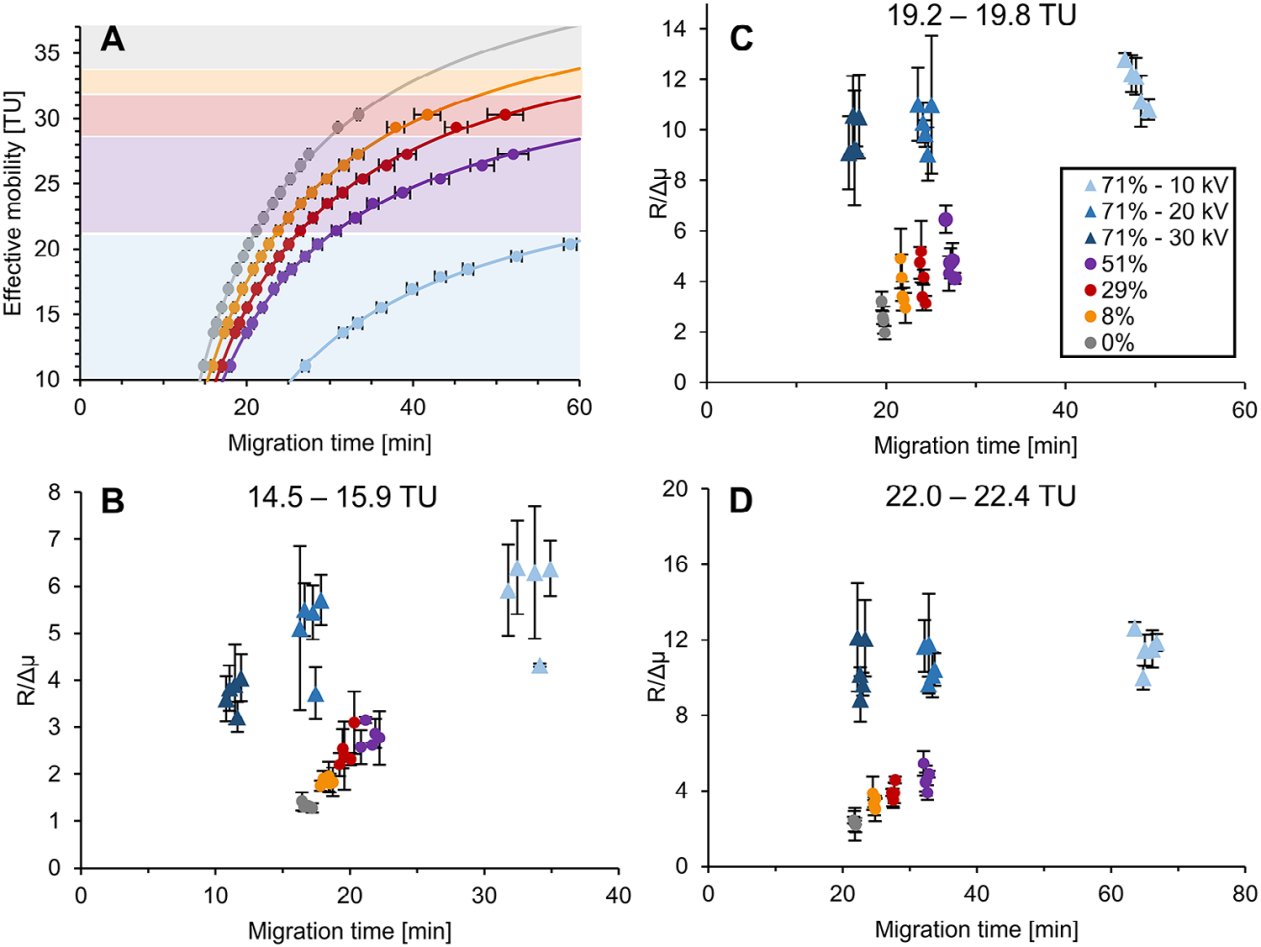
Yeast protein extract separated on PSPSL*_X_*_%_ capillaries (*X* = 0% [gray], 8% [orange], 29% [red], 51% [purple], and 71% [blue], 60 cm each) to show the correlation between A) migration time and effective mobility (17 selected analytes, compare effective mobilities Table S6) on capillaries with different EOF mobilities and B–D) migration time and resolution (*R*/Δ*µ*) on capillaries with different EOF mobilities and at different voltages (−10 kV [light blue], −20 kV [middle blue], and −30 kV [dark blue]) for five selected analytes per panel (Table S10) with a B) low analyte mobility (14.5–15.9 TU), C) medium analyte mobility (19.2–19.8 TU), and D) medium‐high analyte mobility (22.0–22.4 TU). Other separation parameters are found in Materials and Methods.

In Figure 6A, the effective mobilities of 17 analytes from the yeast protein extract are plotted against their migration time for 0%, 8%, 29%, 51%, and 71% sulfobetaine‐modified α‐PLL coating. Increasing the modification degree increases both the migration times of the analytes and the migration time differences between each of the analytes.

Figure 6B–D shows the relationship between the resolution of peaks, the used coating, and applied voltage for three different mobilities using five analytes each. As a normalized value, the resolution is calculated per 1 TU (*R*/Δ*µ*) (see Figure S12 for more details). When comparing the same analytes on different coatings at a voltage of −10 kV (Figure 6B), there is a clear trend of higher resolution values for the coatings with a lower EOF strength (e.g., PSPSL_71%_) and lower resolution values for the coatings with a higher EOF strength (e.g., PSPSL_0%_). The same result can be observed for analytes of higher mobility (Figure 6C,D). Of course, the migration time increases with lower EOF, but it can be reduced by increasing the separation voltage keeping a better resolution at comparable migration time (see e.g., Figure 6C,D, dark blue vs. gray). This is only possible for capillary coatings leading to relatively flat plate height *H* vs. solute migration velocity *u* curve. This can be obtained in the case of relatively homogeneous surface charge coating [44].

Following Equation (1), the correlation between migration time and resolution is linear as long as the separation efficiency of the proteins for the coatings does not change. In Figure S13, a trendline is fitted through the data points of the individual analytes (same Figure as Figure 6B–D colored according to individual analytes). The analytes roughly fit the trendline with the resolution values lower than expected for the higher EOF coatings.

For analytes with even higher mobilities, other coatings are required; for example, for analytes with mobility of around 30 TU, the PSPSL_51%_ is the best choice.

Although the reduced EOF leads to the highest resolution, the analysis time becomes quite high (>3 h for the 180 cm long capillary). In proteomics, longer migration time frames can be an advantage as they allow for more MS/MS experiments. Nevertheless, for other applications, shorter migration times might be desired. Here, still, the modified polylysine coatings can be advantageous as combining the PSPSL_71%_ coating with a high voltage results in higher resolution and similar migration times than the PSPSL_0%_ coating at a lower voltage.

## 4 Concluding Remarks

In this work, zwitterionic modified α‐PLL coatings are used to increase the resolution of proteoforms in several model proteins as well as intact proteins and proteoforms in a yeast protein extract. The selective modification of the lysine side chains in the α‐PLL coating with the zwitterionic sulfobetaine functionality allows for the adjustment of the EOF strength depending on the modification degree of the coating. Using absolute mobilities of the EOF that are similar to the effective mobility of the analyte allows for a strong increase in peak resolution. When comparing a high EOF and a medium EOF coating, it becomes clear that if all analytes should be separated using the same capillary, a high EOF coating should be used, while a coating exhibiting a lower EOF strongly increases the peak resolution and the number of detected proteoforms for the covered mobility range. Combining the analysis on two coatings of different mobility, a much higher number of analytes in the proteomic sample can be identified. Additionally, using coatings with a freely selectable EOF will allow for the separation of critical analytes in various applications. When sequence information and PTMs of a target protein are known, predicted mobility based on mass and net charge can be calculated to choose the right coating (EOF mobility) that results in the best resolution in a given time range. In this way, the here presented medium EOF coatings are ideally suited for the separation of large proteins and their proteoforms with a low positive net charge such as proteins with high amounts of acidic PTMs like phosphorylation, glycosylation, and acetylation (reducing the number of basic functionalities).

## Conflicts of Interest

The authors declare no conflicts of interest.

## Supporting information




**Supporting File 1**: pmic70012‐sup‐0001‐SuppMat.xlsx

**Supporting File 2**: pmic70012‐sup‐0002‐SuppMat.docx

## Data Availability

All CZE‐MS/MS raw data have been uploaded to ProteomeXchange Consortium via the PRIDE partner repository and are available with the identifier PXD061793.
