# Supplementary material for: Enhanced Separation of Intact Proteins and Proteoforms by CZE‐MS Using Sulfobetaine‐Modified Poly(α‐L‐lysine)‐Based Multilayer Coatings for EOF Adjustment
Source: Proteomics. 2025 Jul 25;25(24):77–87. doi: 10.1002/pmic.70012 (PMC12716118; doi:10.1002/pmic.70012)
Supplement: Supplementary file 2 — Supporting File 2: pmic70012‐sup‐0002‐SuppMat.docx [file PMIC-25--s002.docx]

**Enhanced separation of intact proteins and proteoforms by CZE-MS using sulfobetaine-modified poly(α-L-lysine)-based multilayer coatings for EOF adjustment**

Alisa Höchsmann^1,2^, Henry Frick^1^, Laura Dhellemmes^3^, Laurent Leclercq^3^, Philipp T. Kaulich^4^, Andreas Tholey^4^, Hervé Cottet^3^, Norbert Schaschke^1^, Christian Neusüß^1^*

^1^ Faculty of Chemistry, Aalen University, Aalen, Germany

^2^ Faculty of Science, University of Tübingen, Tübingen, Germany

^3^ IBMM, University of Montpellier, CNRS, ENSCM, Montpellier, France

^4^ Systematic Proteome Research & Bioanalytics, Institute for Experimental Medicine, Christian-Albrechts-Universität zu Kiel, Kiel, Germany

***Correspondence:** Prof. Dr. Christian Neusüß, Beethovenstr. 1, 73430 Aalen, Germany, Christian.Neusuess@hs-aalen.de

Table of Contents

[1 Sulfobetaine-modified poly(α-L-lysine) 3](#_Toc200891858)

[Figure S1. 3](#_Toc200891859)

[2 Additional Methodological Information 4](#_Toc200891860)

[Table S1 4](#_Toc200891861)

[3 Separation of model proteins 5](#_Toc200891862)

[Table S2 5](#_Toc200891863)

[Table S3 5](#_Toc200891864)

[3.1 Fetuin 6](#_Toc200891865)

[Figure S2 6](#_Toc200891866)

[Figure S3 7](#_Toc200891867)

[3.2 Casein 8](#_Toc200891868)

[Figure S4 8](#_Toc200891869)

[Table S4 9](#_Toc200891870)

[Figure S5 10](#_Toc200891871)

[4 Sample Preconcentration 11](#_Toc200891872)

[Figure S6 11](#_Toc200891873)

[Figure S7 12](#_Toc200891874)

[Figure S8 12](#_Toc200891875)

[5 Calculation of effective mobility from unknown analytes 13](#_Toc200891876)

[Table S5 13](#_Toc200891877)

[Figure S9 14](#_Toc200891878)

[Table S6 15](#_Toc200891879)

[Figure S10 15](#_Toc200891880)

[Table S7 16](#_Toc200891881)

[Table S8 16](#_Toc200891882)

[6 Separation Yeast Sample 17](#_Toc200891883)

[Figure S11 17](#_Toc200891884)

[Table S9 17](#_Toc200891885)

[7 Calculation of resolution R/Δµ 18](#_Toc200891886)

[Figure S12 18](#_Toc200891887)

[Figure S13 19](#_Toc200891888)

[Table S10 20](#_Toc200891889)

# 1 Sulfobetaine-modified poly(α-L-lysine)


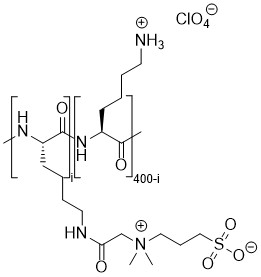


Figure S1. Structure of sulfobetaine-modified α-PLL used as outermost layer (assigned as L_X%_ where X indicates the degree of functionalization) in the SMIL coatings. i ranging from 32 - 284 indicates the composition of the randomly derivatized α-PLL (L_8%_: i = 32, L_29%_: i = 116, L_51%_: i = 204, and L_71%_: i = 284).

# 2 Additional Methodological Information

Table S1: MS Parameters.

| **Sample** | **Intact Yeast**  **Casein** | **Fetuin, AGP, BSA** |
| --- | --- | --- |
|  |  |  |
| Spray voltage | 2000 V (Positive) | 2000 V (Positive) |
| Sweep Gas (Arb) | 3 | 3 |
| Ion Transfer Tube Temp | 300 °C | 300 °C |
| Application Mode | Intact Protein | Intact Protein |
| Pressure Mode | Low Pressure | Low Pressure |
| **Scan Parameters** |  |  |
| Detector Type | Orbitrap | Orbitrap |
| Orbitrap Resolution | 120 000 | 15 000 |
| Scan range (m/z) | 400 – 2000 | 1000 – 3500 |
| Maximum Injection Time | 250 ms | 50 ms |
| Normalized AGC Target | 200% | 100% |
| Microscans | 4 | 15 |
| RF Lens | 30% | 90% |
| Source Fragmentation Energy | 15 V | 60 V |
| **Dynamic Exclusion** |  | no MS/MS |
| Exclusion after n times | 2 |  |
| Exclusion duration (s) | 60 |  |
| Mass Tolerance | mz, 1.5 |  |
| Exclude isotopes | True |  |
| Perform dependent scan on single charge state per precursor only | False |  |
| **Filter Charge State** |  |  |
| Include charge states | 4-50 |  |
| Include undetermined charge states | True |  |
| **ddMSn Scan** |  |  |
| MSn Level | 2 |  |
| Desired minimum points across the peak | 6 |  |
| Isolation Mode | Quadrupole |  |
| Isolation Window | 5 |  |
| Activation Type | CID |  |
| Collision Energy | 30% |  |
| Activation Time | 10 ms |  |
| Activation Q | 0.25 |  |
| Detector Type | Orbitrap |  |
| Orbitrap Resolution | 60 000 |  |
| Scan Range | 150 – 2000 |  |
| Maximum Injection time | 250 ms |  |
| Normalized AGC Target | 1000% |  |
| Microscans | 4 |  |

# 3 Separation of model proteins

Table S2: Overview of separation conditions for the proteins shown in Figure 1.

| **Figure 1** | **Analyte** | **Smoothing** | **Coating**  (Last Layer) | **m/z**  (for BPE) | **Separation**  **voltage** | **Injection volume** |
| --- | --- | --- | --- | --- | --- | --- |
| **A** | Fet AGP | 0 | PDADMAC | 1000-3500 | -10 kV | 7 s, 33 mbar |
| **B** | Fet AGP | Gaussian 5 | Polylys-71% | 1000-3500 | -10 kV | 7 s, 33 mbar |
| **C** | BSA | 0 | PDADMAC | 1000-3500 | -10 kV | 7 s, 33 mbar |
| **D** | BSA | Gaussian 5 | Polylys-51% | 1000-3500 | -10 kV | 7 s, 33 mbar |
| **E** | mAb | 0 | PDADMAC | 700-4000 | -20 kV | 5 s, 40 mbar |
| **F** | mAb | Gaussian 5 | Polylys-71% | 700-4000 | -10 kV | 5 s, 40 mbar |
| **G** | Casein | 0 | PDADMAC | 800-2000 | -10 kV | 7 s, 33 mbar |
| **H** | Casein | Gaussian 5 | Polylys-71% | 800-2000 | -10 kV | 7 s, 33 mbar |

Table S3: Resolution values for two selected species of Fetuin, AGP and Casein calculated for the PSPSL_71%_ and the PSPSP coating (each n=3).

|  | Fetuin | AGP | Casein |
| --- | --- | --- | --- |
| Used m/z | 2159.22  2052.71 | 2561.83  2514.95 | 1091.20  1092.97 |
| PSPSP, -10 kV | R= 0.42 ± 0.09 | R = 0.90 ± 0.02 | R = 0.70 ± 0.07 |
| PSPSL_71%_, -10 kV | R = 0.94 ± 0.05 | R = 1.54 ± 0.06 | R = 1.92 ± 0.18 |
| PSPSL_71%_/ PSPSP | 2.2 | 1.7 | 2.7 |

# 3.1 Fetuin

Fetuin is a glycoprotein with proteoforms ranging in size between 42 kDa to 49 kDa, mainly depending on the amount of glycosylation. Due to the high amount of different glycoforms, no complete separation was achieved, but in general, larger mass analytes can be found at the beginning of the peaks, while lower mass analytes can be found for higher migration times (Figure S2). Prominent mass shifts in between the detected main forms are + 291 Da, the mass difference of one additional sialic acid, + 365 Da, the mass difference of an additional antenna (HexNac + Hex), and + 80 Da, the mass difference of one additional phosphate. (Figure S3).


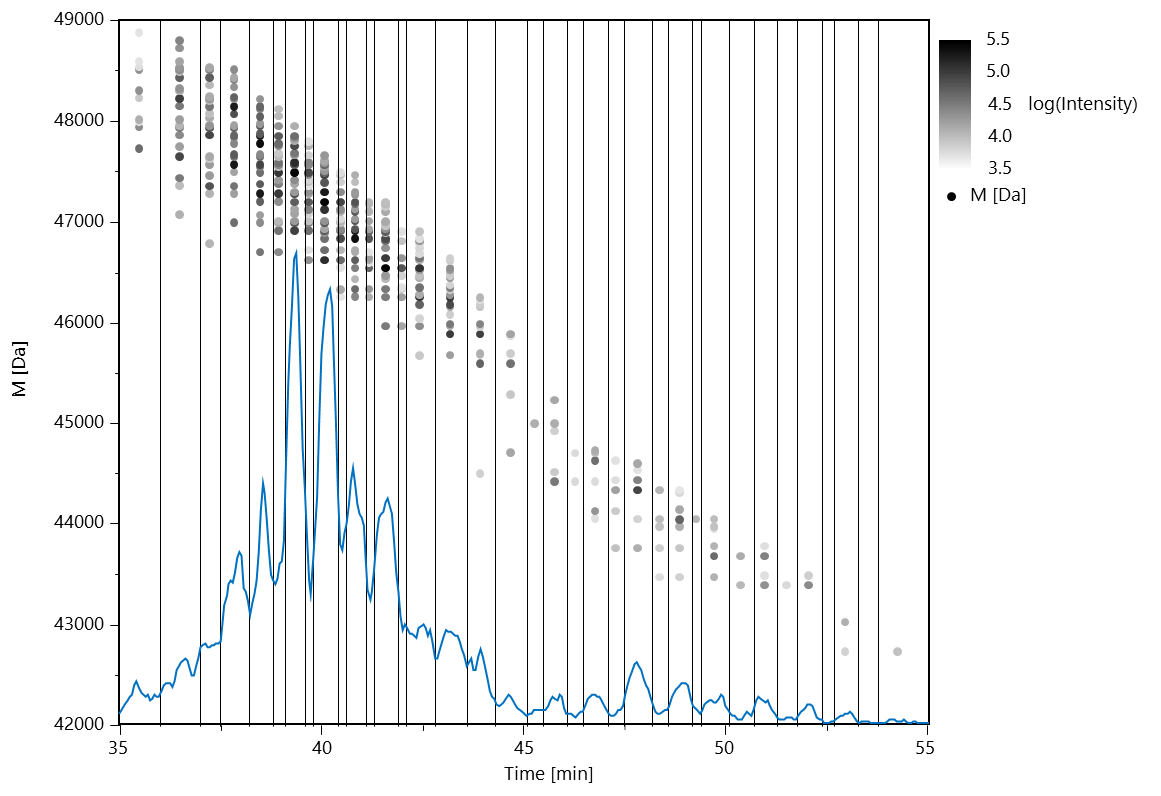


Figure S2: Electropherogram of Fetuin on the scale of migration time against detected molecular masses M in Da (with marked slices and the 20 highest intense deconvoluted masses (above 5x10^3^ intensity) found in each slice (decided based on the shape of the base peak electropherogram), masses obtained by Protein Metrics (deconvoluted mass range: 10000 – 50000, auto mass peak picking: min difference between mass peaks 15 Da, max number of mass peaks: 20, no peak sharpening)).


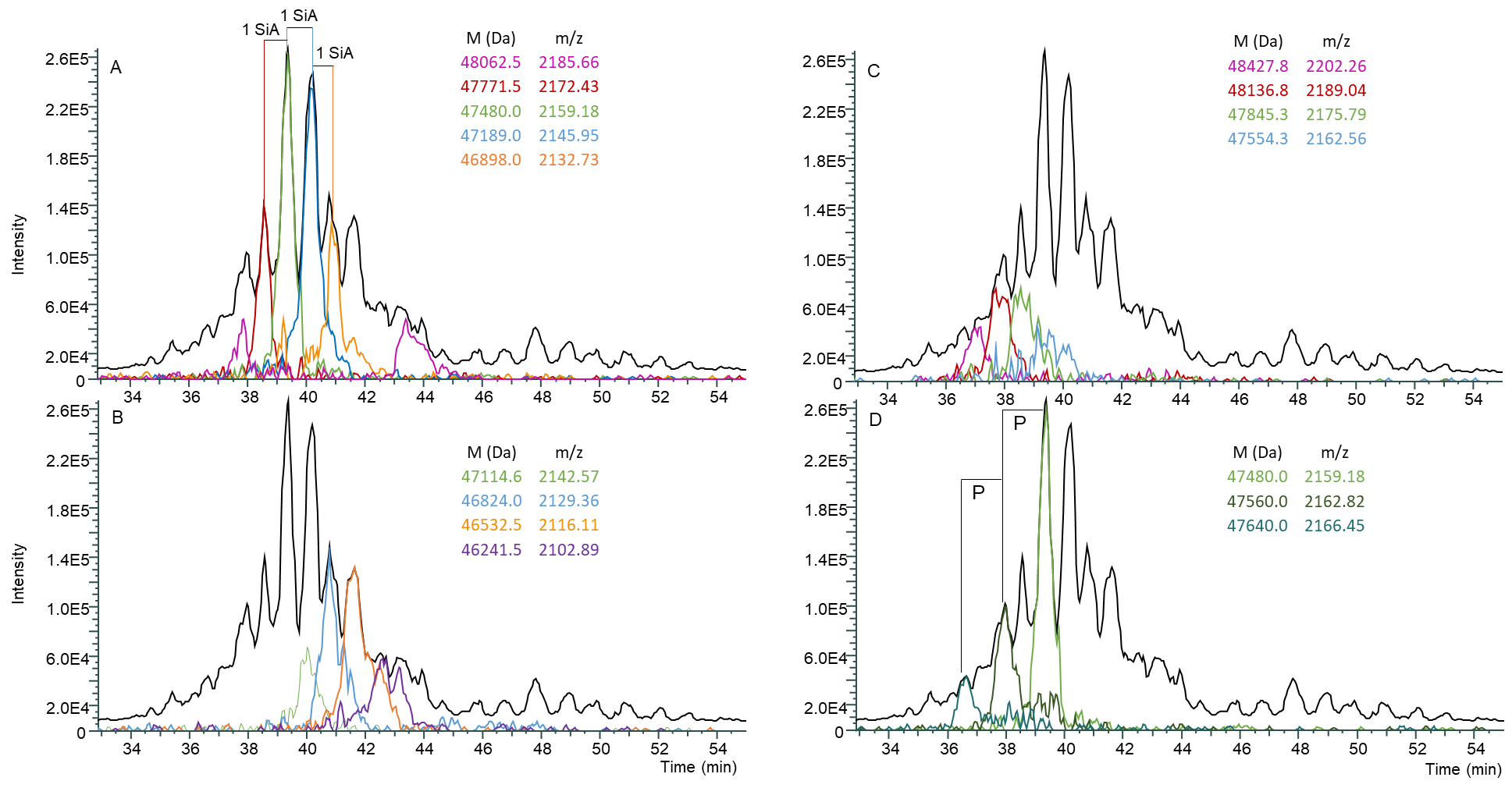


Figure S3: Electropherogram of certain high intense mass shifts detected in the fetuin peak. A,B,C) Extracted ion electropherograms (EIEs) are plotted based on the *m/z* found in the small table with a mass shift of ~291 Da in between each of the deconvoluted masses (obtained by Protein Metrics). In between EIEs of the same color for A-C) mass difference of ~ 365 Da. D) Depiction of EIEs on m/z-base with a mass shift of ~80 Da in between their deconvoluted masses, EIE depicted: m/z with ± 0.3 Da.

# 3.2 Casein


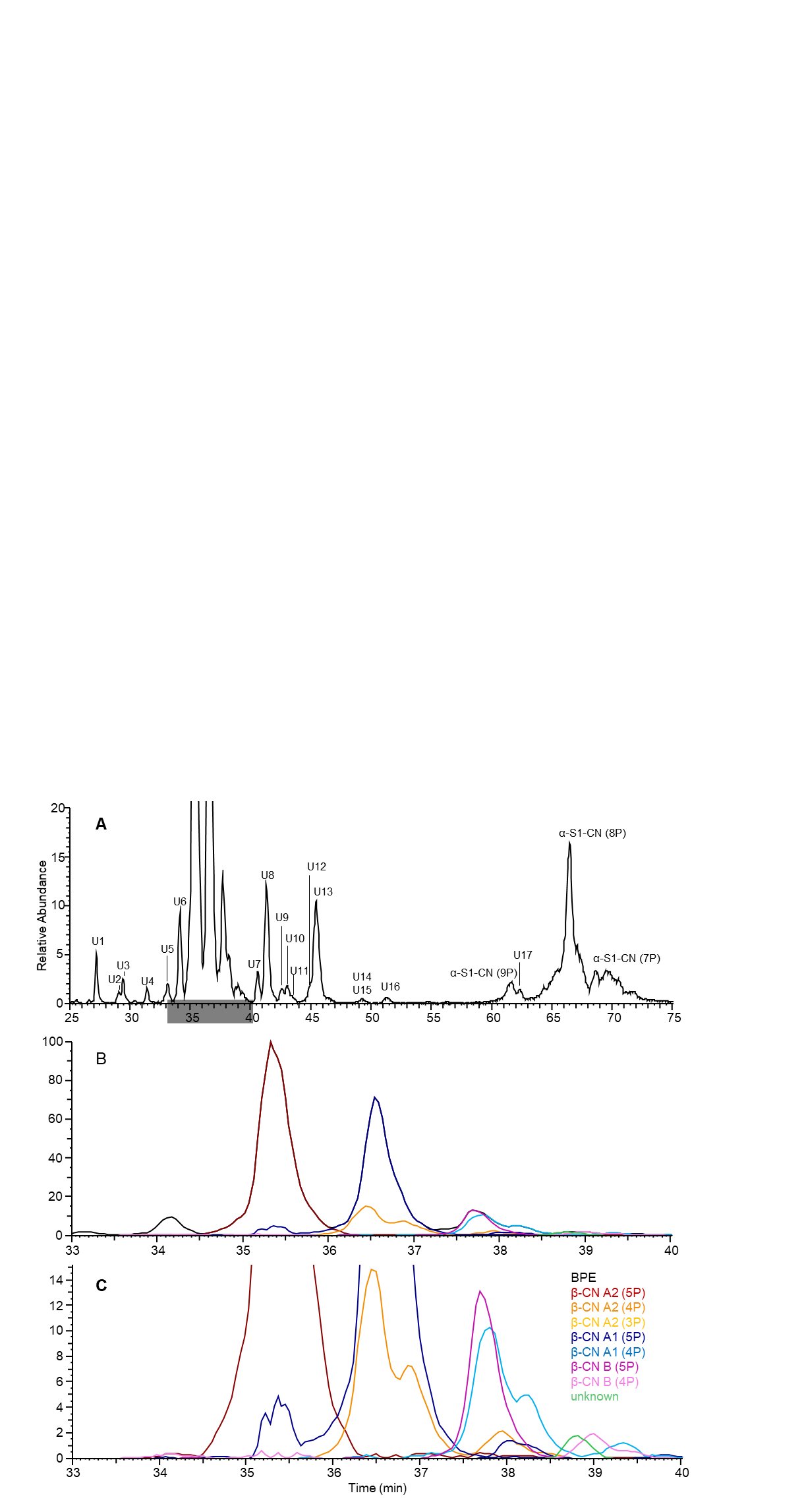


Figure S4: Separation of proteoforms of Casein using the PSPSL-71% coating. Whole electropherogram deconvoluted by Xtract (default values). A) Deconvoluted base peak electropherogram, B) Zoom on the x-axis (33 min – 40 min) to display the β-Casein forms and C) Zoom on the x-axis as in B and y-axis (0-15% Relative Abundance). Both (B+C), deconvoluted extracted ion electropherogram for the different colored species with their deconvoluted mass in Table S4 (for display; M with ± 2 Da). Further separation conditions are listed in Table S2 and Material and Methods.

Table S4: High intense masses found in the sample of Casein as shown in Figure S4.

| Protein | Phosphoryl-ation | Measured Mass  [Da] | Calculated Mass^a^  [Da] | Relative  Peak Area^b^  [%] | Confirmed  with MS/MS^a^ |
| --- | --- | --- | --- | --- | --- |
| β-CN A2 | 5 P | 23969.0 | 23968.2 | 100 | Yes |
| β-CN A2 | 4 P | 23889.3 | 23888.2 | 19.8 ± 0.5 | Yes^c^ |
| β-CN A2 | 3 P | 23809.7 | 23808.2 | 1.3 ± 0.1 | Yes |
| β-CN A1 | 5 P | 24009.3 | 24008.2 | 67.8 ± 0.5 | Yes |
| β-CN A1 | 4 P | 23929.3 | 23928.2 | 13.6 ± 0.1 | Yes^c^ |
| β-CN B | 5 P | 24078.3 | 24077.2 | 11.9 ± 0.1 | Yes |
| β-CN B | 4 P | 23998.3 | 23997.3 | 1.8 ± 0.1 | Yes^c^ |
| unknown |  | 22827.8 |  | 1.2 ± 0.1 |  |
| U1 |  | 12171.0 |  |  |  |
| U2 |  | 12091.0 |  |  |  |
| U3 |  | 12210.0 |  |  |  |
| U4 |  | 12436.1 |  |  |  |
| U5 |  | 24916.6 |  |  |  |
| U6 |  | 11533.1 |  |  |  |
| U7 |  | 20510.8 |  |  |  |
| U8 |  | 15764.5 |  |  |  |
| U9 |  | 20550.8 |  |  |  |
| U10 |  | 15225.2 |  |  |  |
| U11 |  | 15834.6 |  |  |  |
| U12 |  | 11829.3 |  |  |  |
| U13 |  | 11816.3 |  |  |  |
| U14 |  | 21863.2 |  |  |  |
| U15 |  | 11885.4 |  |  |  |
| U16 |  | 12670.8 |  |  |  |
| U17 |  | 14750.3 |  |  |  |
| α-S1-CN | 9 P | 23680.3 | 23680.2 |  |  |
| α-S1-CN | 8 P | 23600.3 | 23600.2 |  |  |
| α-S1-CN | 7 P | 23521.3 | 23520.3 |  |  |

^a^ Monoisotopic mass and information about PTMs were obtained using information from the UniProtKB database (http://www.uniprot.org) and using ProSight Light (http://prosightlite.northwestern.edu/)

^b^ Relative to Peak Area of β-CN A2, unseparated peaks like e.g. β-CN A2 4P are integrated as one, mean values and standard deviation calculated on three repeats

^c^ position of missing phosphorylation unclear


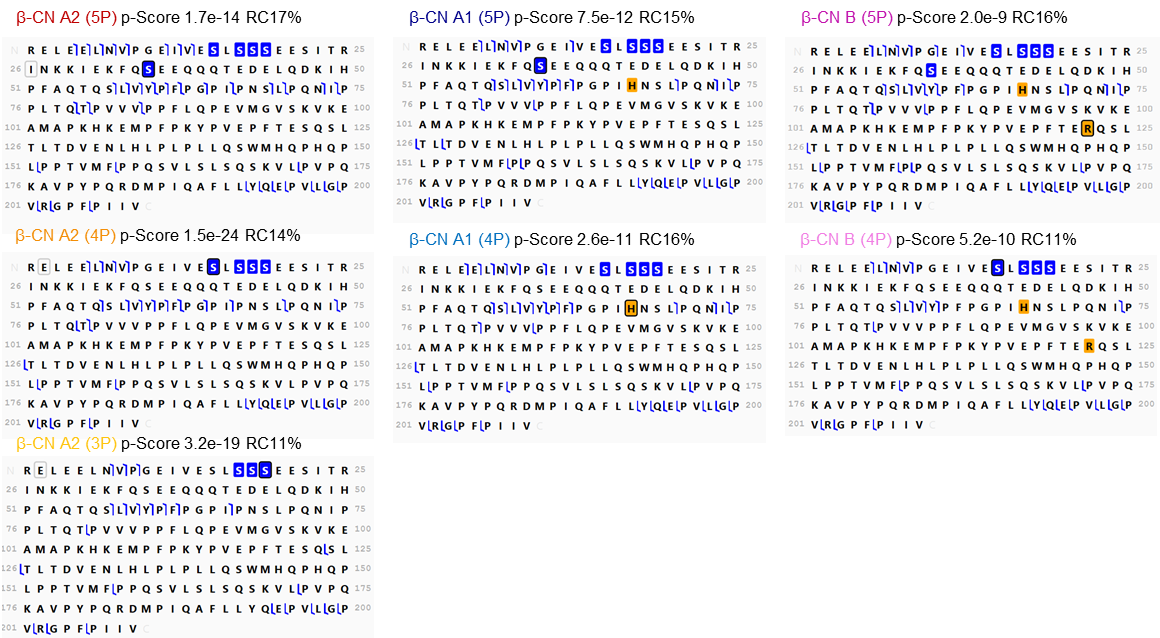


Figure S5: Fragmentation maps of the proteoforms of β-Casein for the peaks detected in Figure S4 and Table S4. Imagines with ProSight Light, RC: Residue Cleavages.

# 4 Sample Preconcentration


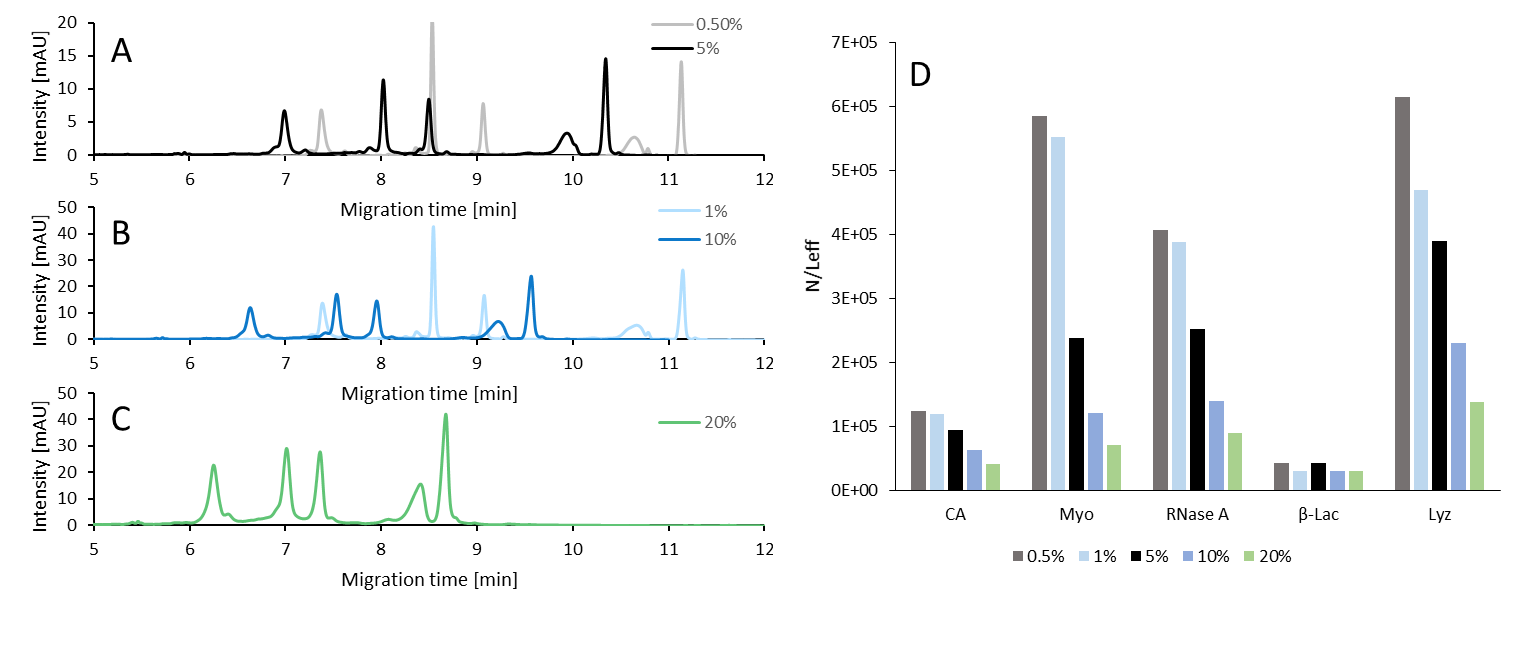


Figure S6: Initial experiments for sample preconcentration where the capillary is filled with different volumes (0.5%: 7 s, 33 mbar, 1%: 14 s, 33 mbar, 5%: 70 s, 33 mbar, 10%: 140 s, 33 mbar, 20%: 280 s, 33 mbar) of sample solution (200 µg/mL (0.5% and 1%) and 20 µg/mL (5%, 10%, 20%) of five proteins (carbonic anhydrase (CA), myoglobin (Myo), ribonuclease A (RNase A), β-lactoglobulin A (β -Lac) and lysozyme (Lyz)) in 50 mM ammonium acetate + 100 mM acetic acid) and separated with a PSPSP coating at -30 kV, A – C comparison of separation for different injection volumes with the same sample concentration on the capillary A) 1.18 ng protein, B) 2.36 ng protein and C) 4.72 ng protein. D) Separation efficiency N/Leff (Leff = 0.515 m) for each of the five injection volumes and each of the five proteins.


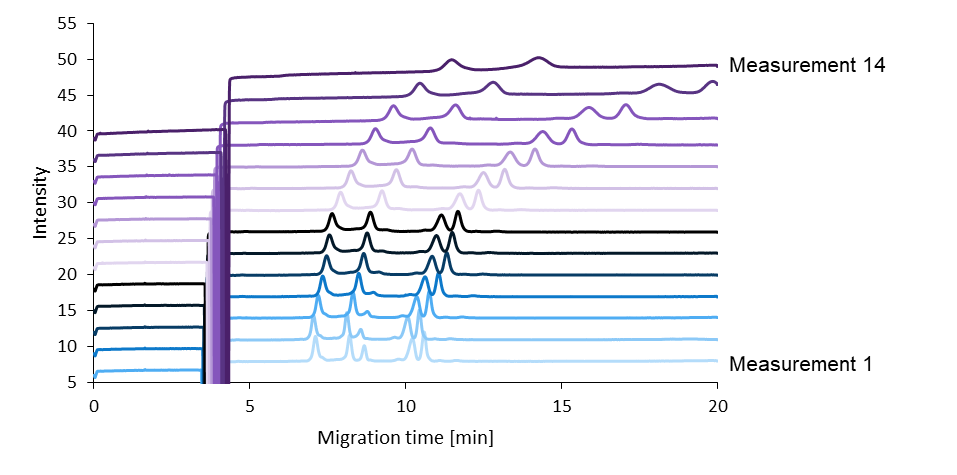


Figure S7: Separation of the five proteins (left to right: CA, Myo, RNase A, β-Lac, Lyz) mixture using the PSPSP coating at -30 kV, with measurements of the yeast sample in between every protein measurement. Injection volume: 7 s, 33 mbar.


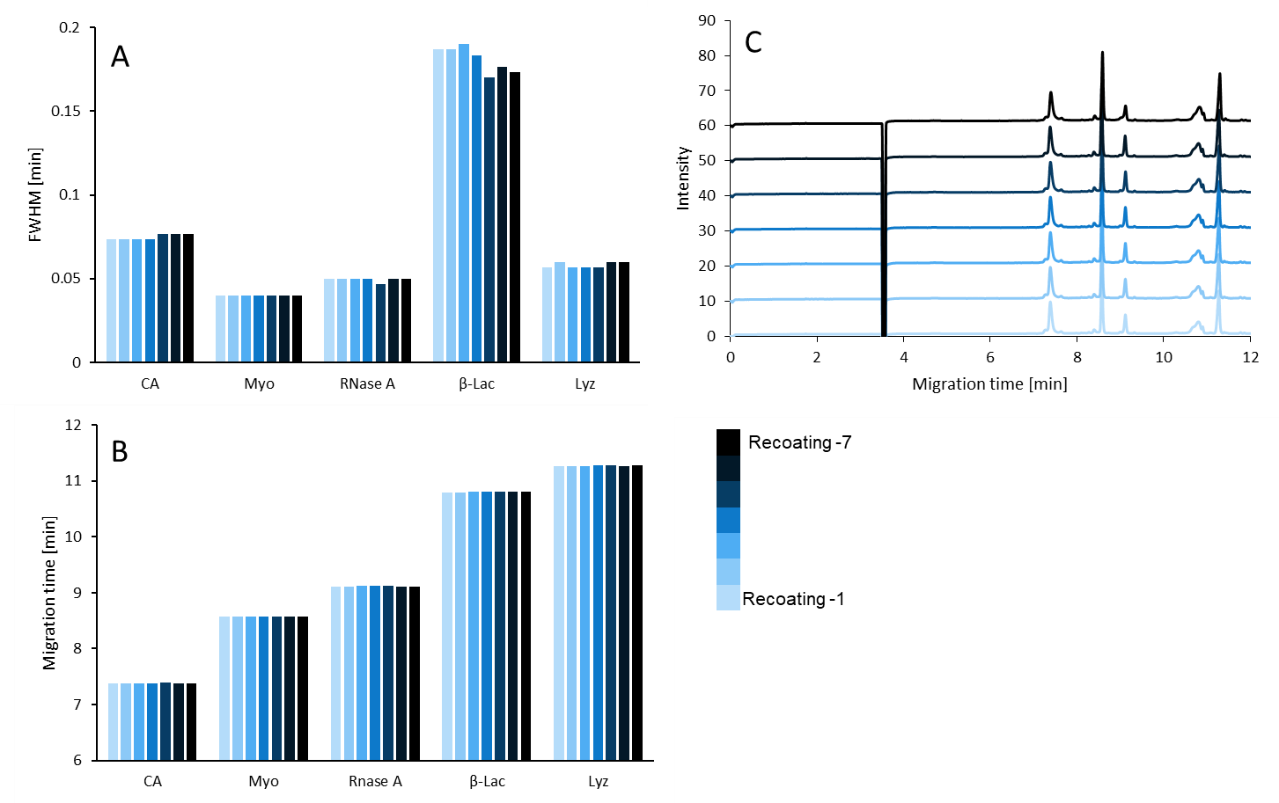


Figure S8: Separation of the five proteins mixture using the PSPSP coating at -30 kV, with measurements of the yeast sample in-between every protein measurement and recoating of the capillary before every protein measurement. A) Change in peak width based on full width at half maximum (FWHM), B) Change in migration time, and C) Electropherograms for seven measurements of the five protein mixture (carbonic anhydrase (CA), myoglobin (Myo), ribonuclease A (RNase A), β-lactoglobulin A (β -Lac) and lysozyme (Lyz)). Injection volume: 7 s, 33 mbar.

# 5 Calculation of effective mobility from unknown analytes

Five standard proteins (Table S5) were spiked into the yeast cell lysate with a concentration of 20 µg/mL. Myoglobin was neglected in further considerations due to the low signal intensity. The sample is measured on the PSPSP capillary six times.

Table S5: List of four standard proteins with µ(analyte) of the four proteins obtained out of previous measurements with the proteins dissolved in BGE separated by CE-UV using 2 M HAc as BGE.

| Protein | Deconvoluted mass M | µ(analyte) [TU] |
| --- | --- | --- |
| Carb | 29005.79,29006.79 | 24.2 |
| RNase | 13673.33 | 28.4 |
| Beta-Lac | 18351.55 | 31.0 |
| Lys | 14295.92 | 31.8 |

The migration time of the four standard proteins was obtained out of the deconvoluted data file. The deconvolution was performed using FreeStyle Xtract Deconvolution (Monoisotopic Masses, Output Mass: M, Adduct Element: H^+^, Charge Range 2-50, Min Num Detected Charge: 2). Afterwards, the extracted ion electropherogram (EIE) of the deconvoluted mass is plotted to gain the migration time of each protein.

In the next step, the effective analyte mobilities of the standard proteins were plotted against their reciprocal migration time (1/t) in jmp and a linear slope was fitted through the four points (Figure S9) that can be used to gain the values for a and b of equation (S1).

$\mu_{e}=a-b \frac{1}{t}$ (S1)

Equation S1 allows for the calculation of all effective mobilites *µ_e_* and the absolute mobility of the EOF without the addition of an EOF marker or model proteins to the sample. Additionally, it considers the slight deviation of the applied CE voltage due to the electrospray voltage (+ 2 kV). When coupling the nanoCEasy interface to the Orbitrap Lumos, the ES voltage is applied to the interface and, therefore, impacts the total electric field along the capillary.


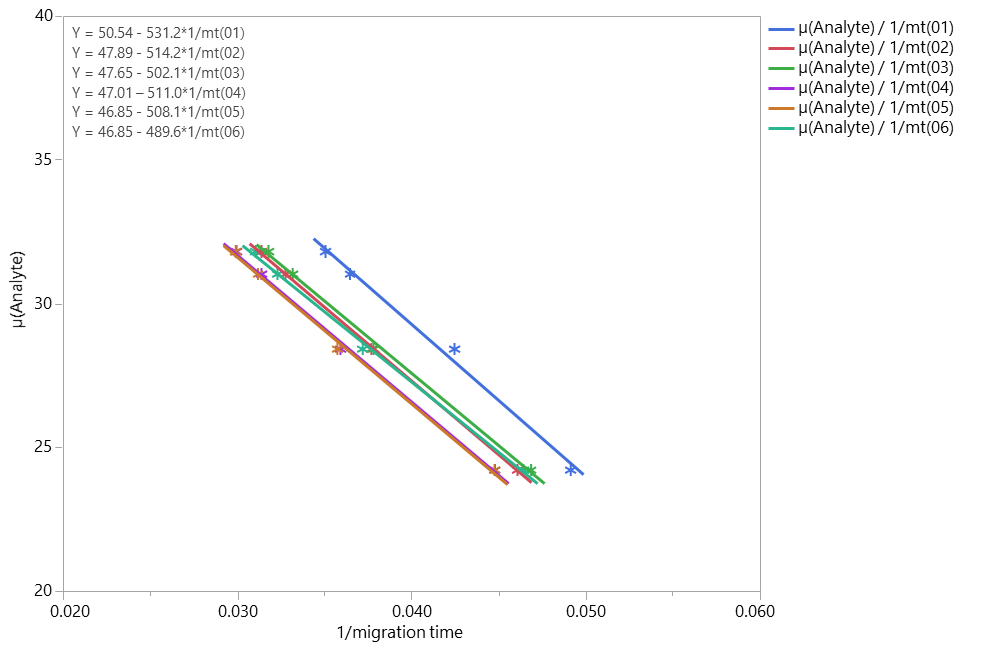


Figure S9: Effective analyte mobilities of the standard proteins (µ(Analyte)) plotted against their reciprocal migration time (1/migration time) in jmp to obtain the values for the slope for six repeat measurements (injection volume: 5% capillary volume, separation voltage: -10 kV, PSPSP coating).

The same linear slope was calculated for each of the six repeat measurements (a and b, RSD <3%). The obtained equation (S1) was used to calculate the effective analyte mobilities for 17 unknown analytes of the yeast sample using their migration time. After the effective mobilities were calculated, a mean of the six values was obtained and used for further measurement (Table S6). The correlation between effective mobility and migration time is shown in Figure S10. Using the effective mobilities for the 17 analytes, equation S1 can be solved for the separation of the yeast sample on other coatings with an unknown EOF, namely all the PSPSL_X%_ coatings.

Table S6: Calculated effective mobilities for 17 analytes found in the yeast sample (n=6) for the PSPSP coating (60 cm).

| m/z | µ_Run1_ [TU] | µ_Run2_  [TU] | µ_Run3_  [TU] | µ_Run4_  [TU] | µ_Run5_  [TU] | µ_Run6_  [TU] | $\bar{\boldsymbol{\mu(Analyte)}}$  (Run1-6) [TU] | RSD  [%] |
| --- | --- | --- | --- | --- | --- | --- | --- | --- |
| 4247.0 | 11.4 | 11.0 | 11.1 | 10.6 | 10.8 | 11.3 | 11.04 | 2.87 |
| 8160.2 | 14.0 | 13.6 | 13.7 | 13.2 | 13.4 | 13.9 | 13.61 | 2.13 |
| 9579.6 | 14.6 | 14.3 | 14.4 | 14.0 | 14.2 | 14.5 | 14.33 | 1.65 |
| 4467.4 | 15.9 | 15.5 | 15.6 | 15.2 | 15.3 | 15.6 | 15.53 | 1.71 |
| 9852.8 | 17.4 | 17.1 | 17.0 | 16.6 | 16.7 | 17.0 | 16.94 | 1.67 |
| 12061.3 | 18.0 | 17.6 | 17.9 | 17.7 | 17.9 | 18.1 | 17.86 | 0.85 |
| 9267.9 | 18.7 | 18.4 | 18.5 | 18.3 | 18.3 | 18.6 | 18.47 | 0.92 |
| 6482.5 | 19.7 | 19.5 | 19.5 | 19.3 | 19.3 | 19.5 | 19.44 | 0.91 |
| 5408.9 | 20.6 | 20.4 | 20.3 | 20.3 | 20.2 | 20.4 | 20.37 | 0.73 |
| 9256.0 | 21.7 | 21.4 | 21.3 | 21.2 | 21.3 | 21.4 | 21.40 | 0.79 |
| 9238.7 | 22.7 | 22.4 | 22.3 | 22.3 | 22.3 | 22.4 | 22.39 | 0.64 |
| 8551.6 | 23.8 | 23.5 | 23.4 | 23.4 | 23.4 | 23.5 | 23.48 | 0.68 |
| 11595.7 | 24.5 | 24.3 | 24.3 | 24.3 | 24.2 | 24.3 | 24.32 | 0.46 |
| 7221.0 | 25.6 | 25.4 | 25.3 | 25.4 | 25.3 | 25.4 | 25.37 | 0.39 |
| 5043.6 | 26.5 | 26.4 | 26.3 | 26.4 | 26.4 | 26.4 | 26.41 | 0.23 |
| 7058.8 | 30.6 | 30.4 | 30.2 | 30.2 | 30.2 | 30.2 | 30.30 | 0.61 |
| 10997.3 | 31.5 | 31.3 | 31.2 | 31.2 | 31.2 | 31.3 | 31.29 | 0.37 |
| a | 50.5 | 47.9 | 47.6 | 47.0 | 46.9 | 46.9 |  |  |
| b | 531 | 514 | 502 | 511 | 508 | 490 |  |  |


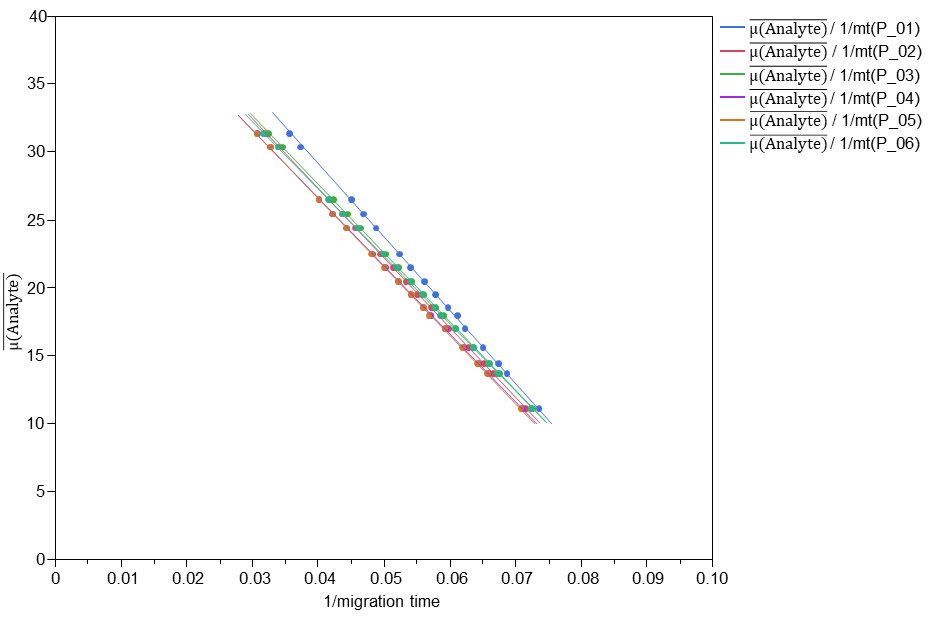


Figure S10: Correlation between the reciprocal migration time and the calculated average effective mobility of 17 yeast analytes for six repeat runs. Figure created in jmp.

In Table S7, equation 1 is solved for the sample preconcentration experiment where different sample volumes (0.5% to 20%) were injected onto the capillary. For larger injection volumes *a* increases while for lower injection volumes *a* decreases, simultaneously there is no clear correlation between the injection volume and *b.*

Table S7: Obtained values for a and b when plotting the migration times from the preconcentration experiments against the µ(analyte) from Table 5.

| Injection volume | a  µ(EOF) | b  constant |
| --- | --- | --- |
| 0.5%: 7 s, 33 mbar | 46.49 | 164.1 |
| 1%: 14 s, 33 mbar | 46.48 | 163.7 |
| 5%: 70 s, 33 mbar | 47.34 | 161.0 |
| 10%: 140 s, 33 mbar | 48.62 | 161.1 |
| 20%: 280 s, 33 mbar | 50.91 | 166.1 |

Using these 17 yeast analytes, the same equation was solved for the 5 different modified polylysine coatings (PSPSL_X%_) (Table S8).

Table S8: Overview of the values for a and b for all coatings PSPSL_X%_ coatings (60 cm); values are based on three repeat measurements on each capillary.

|  | a | b |
| --- | --- | --- |
| 0% | 45.7 ± 0.1 | 513 ± 3 |
| 8% | 41.2 ± 0.4 | 490 ± 2 |
| 29% | 39.8 ± 0.5 | 486 ± 6 |
| 51% | 35.8 ± 0.3 | 443 ± 5 |
| 71% | 28.4 ± 0.3 | 467 ± 18 |

# 6 Separation Yeast Sample


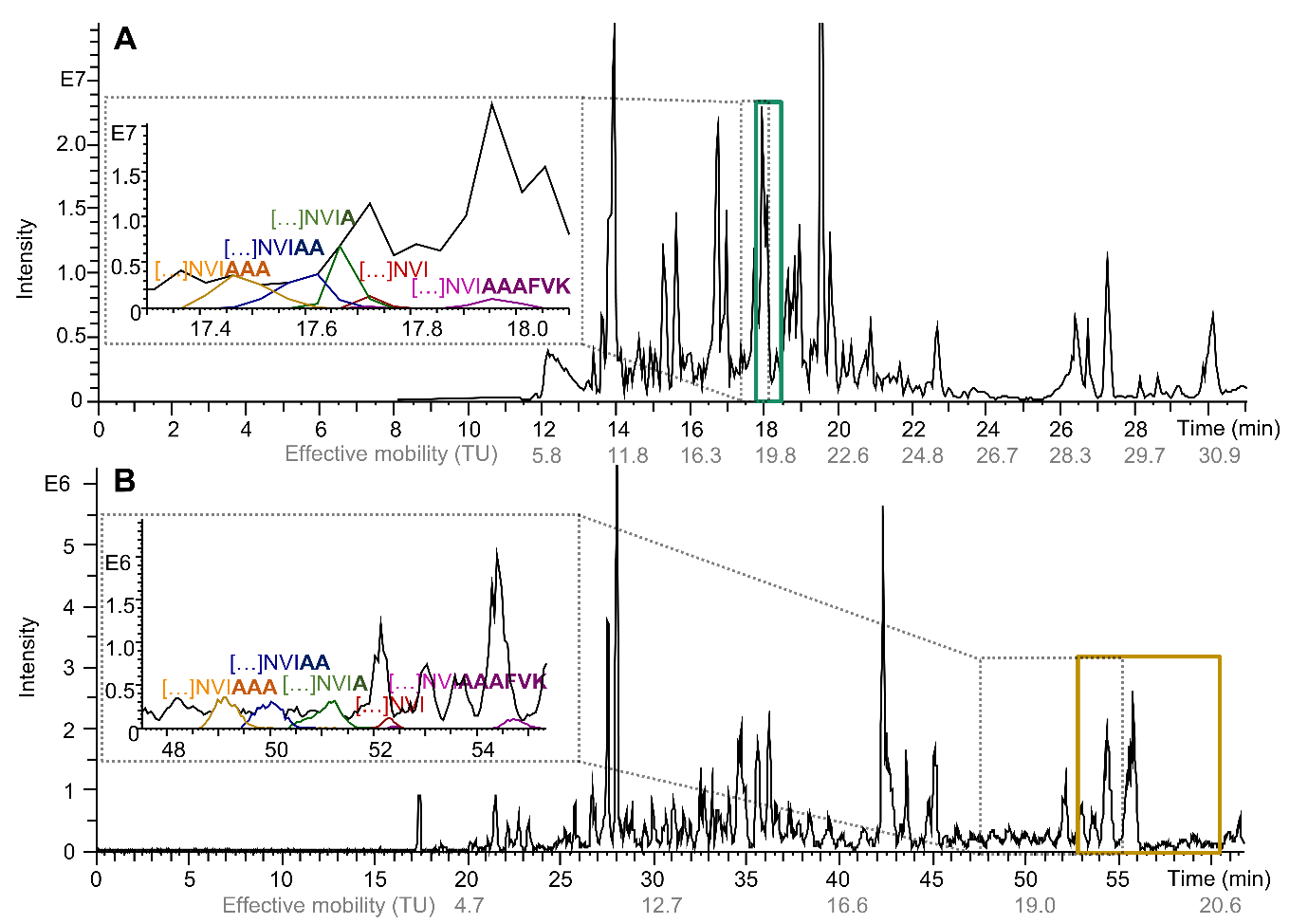


Figure S11: Comparison of the separation of the yeast protein extract for the high (A, PSPSP) and medium EOF (B, PSPSL71%) coating. Deconvoluted Base Peak Electropherogram in black. Dashed insert: Separation of selected truncated forms (different colors, Table S9) of Enolase 2. Solid insert: Analytes with mobilities between 19.5 TU and 20.5 TU, which are used later for Figure 5B. 60 cm capillary length, -10 kV separation voltage, other parameters can be found in the Materials and Methods section.

Table S9: Sequences and intact masses of the analytes depicted in Figure S11 and Figure 3.

|  | Sequence | | M [Da] |
| --- | --- | --- | --- |
| Constant Sequence | [SVYDSRGNPTVEVELTTEKGVFRSIVPSGASTGVH  EALEMRDEDKSKWMGKGVMNAVNNVN]… | |  |
| Yellow | …NVIAAA | 7160.54 ± 0.2 | |
| Blue | …NVIAA | 7089.50 ± 0.2 | |
| Green | …NVIA | 7018.47 ± 0.2 | |
| Red | …NVI | 6947.43 ± 0.2 | |
| Magenta | …NVIAAAFVK | 7534.77 ± 0.2 | |

# 7 Calculation of resolution R/Δµ

The resolution of two peaks depends on their migration time and peak width. Depending on how close the migration times of the selected peak pairs are, the resolution can change drastically. To allow a simple comparison of different resolution values here, R/Δµ is used. This value is the calculated resolution between a measured real peak and its measured peak width and a second simulated peak with a clearly defined lower migration time that has the same peak width as the first peak:

$\frac{R}{\Delta\mu}=1.18 *\frac{t_{\mu} -t_{\mu-1}}{2 *{FWHM}_{\mu}}$ (S2)

With $t_{\mu-1}=\frac{b}{\frac{b}{t_{\mu}}+1}$ (S3)

With $t_{\mu}$ being the migration time of a real peak and $t_{\mu-1}$being the migration time of a fictional peak with an effective mobility µ that is 1 TU smaller than the peak at $t_{\mu}$. ${FWHM}_{\mu}$ is the measured full-width half maximum of the peak at $t_{\mu}$. b is the same named value calculated by equation (1).

When the FWHM of the peaks decreases, fewer points are detected over each peak making obtained information from such peaks less reliable.


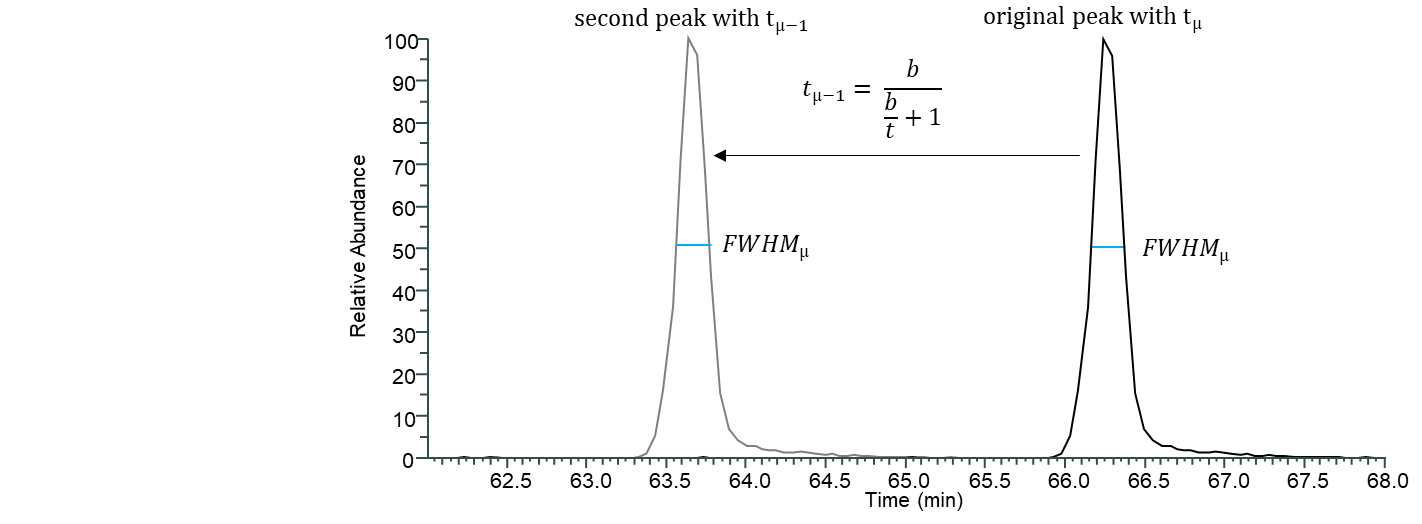


Figure S12: Symbolic depiction of the calculation of R/Δµ.


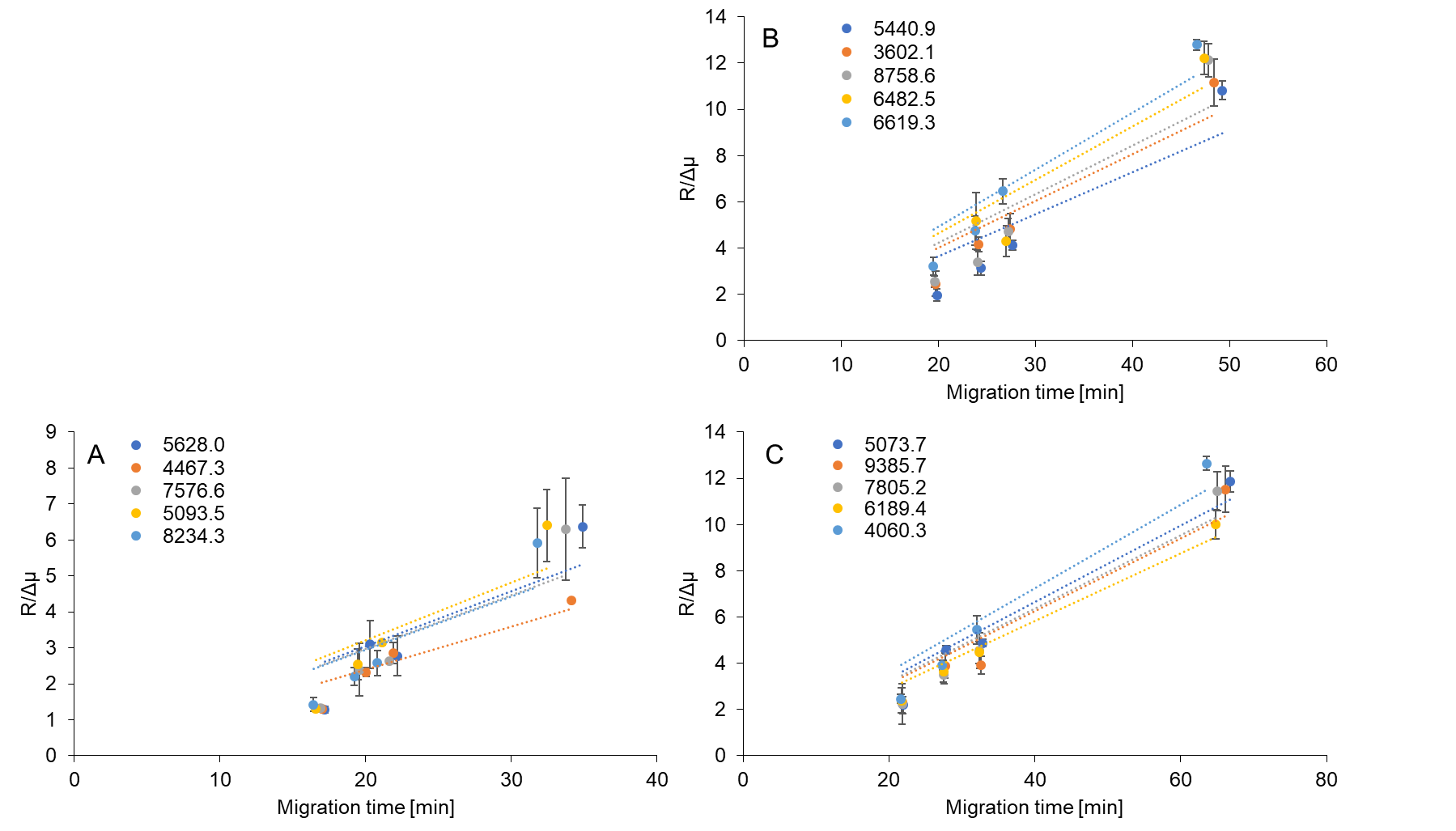


Figure S13: Migration time vs Resolution (R/Δµ) for 15 different analytes (deconvoluted mass, different colors) for the five PSPSL_X%_ coatings. Same analytes as depicted in Figure 6. Trendlines are forced through the origin.

Table S10: Analytes used to calculate the resolution for Figure 6 and S13. Analytes were selected based on their intensity and effective mobility (five analytes in a similar range), Extracted Ion Electropherograms based on the m/z with an allowed difference of 5 ppm were exported and integrated using CEval to obtain the migration time and FWHM.

| M [Da] | *m/z* | µ_e_^a^ |
| --- | --- | --- |
| 5073.7 | 847.12 | 22.4±0.03 |
| 9385.7 | 940.18 | 22.3±0.03 |
| 7805.2 | 868.58 | 22.2±0.03 |
| 6189.4 | 885.49 | 22.1±0.03 |
| 4060.3 | 813.46 | 22.0±0.03 |
| 5440.9 | 908.16 | 19.8±0.01 |
| 3602.1 | 902.03 | 19.7±0.02 |
| 8758.6 | 797.61 | 19.5±0.02 |
| 6482.5 | 927.51 | 19.4±0.02 |
| 6619.3 | 1104.90 | 19.2±0.05 |
| 5628.0 | 1127.21 | 15.9±0.03 |
| 4467.3 | 894.88 | 15.5±0.03 |
| 7576.6 | 1083.81 | 15.4±0.01 |
| 5093.5 | 1020.31 | 14.8±0.03 |
| 8234.3 | 1178.05 | 14.5±0.02 |

^a^ effective mobility calculated based on equation S1 for the 9 measurements (n = 3, voltage: -10 kV, -20 kV, -30 kV) of the PSPSL_71%_ coating ± standard deviation
